# The burden of systemic therapy administration route in treating HER2-positive breast cancer (for patients, healthcare professionals, and healthcare system): a systematic literature review

**DOI:** 10.3389/fphar.2024.1338546

**Published:** 2024-08-19

**Authors:** Luciana Castro Garcia Landeiro, Tamie de Camargo Martins, Ruth Bartelli Grigolon, Isabel Monteiro, Joana Bisol Balardin, Eduardo Padilha, Gilberto Amorim, Stephen Stefani

**Affiliations:** ^1^ Oncology, Oncoclínicas Group, Bahia, Brazil; ^2^ Roche Pharmaceuticals, São Paulo, Brazil; ^3^ Oracle Life Sciences, São Paulo, Brazil; ^4^ Oncology, Instituto D’Or de Pesquisa e Ensino (IDOR), Rio de Janeiro, Brazil; ^5^ Oncology, Núcleo de Avaliação de Tecnologias UNIMED Central, São Paulo, Rio Grande do Sul, Brazil

**Keywords:** HER2, breast cancer, trastuzumab, pertuzumab, subcutaneous administration, intravenous administration

## Abstract

**Introduction:**

Breast cancer (BC) is one of the leading causes of cancer and is the first cause of death from malignant tumors among women worldwide. New cancer therapies receive regulatory approval yearly and to avoid health disparities in society, the health systems are challenged to adapt their infrastructure, methodologies, and reimbursement policies to allow broad access to these treatments. In addition, listening to patients’ voices about their therapy preferences is essential. We aim to investigate the administration route preferences [subcutaneous (SC) or intravenous (IV)] among patients diagnosed with HER2 positive BC and healthcare professionals (HCPs) and to investigate healthcare resources utilization (quality and quantity) for each route of administration (SC or IV) for treating those patients.

**Methods:**

We conducted a systematic literature review focused on clinical trials and observational and economic studies, using PubMed (MEDLINE), Cochrane Library, Virtual Health Library (VHL), Scientific Electronic Library Online (SciELO), and Latin American and Caribbean Health Sciences Literature (LILACS) databases based on the Preferred Reporting Items for Systematic Reviews and Meta-Analysis (PRISMA) statement.

**Results:**

The literature review included 25 studies in the analysis. Studies have reported that patients and HCPs prefer the SC route of administration to IV because it saves time in terms of chair time, administration, and preparation and is less painful. In addition, SC administration might be a more cost-saving option when analyzing direct and indirect costs.

**Discussion:**

As BC stands as a significant global health concern and the leading cause of cancer-related deaths in women worldwide, understanding and incorporating patient and HCPs preferences in the choice of administration route become paramount. The observed preference for SC administration not only aligns with the imperative of adapting health systems to facilitate broad access to new cancer therapies but also underscores the importance of considering patient experiences and economic implications in shaping treatment strategies. These insights are crucial for healthcare policymakers, clinicians, and stakeholders in optimizing healthcare resources and enhancing the overall quality of BC care.

## 1 Introduction

Breast cancer (BC) is one of the leading causes of cancer among women worldwide, accounting for 15% of new annual female cancer cases (GBD, 2017 Causes of Death Collaborators, 2018; GBD, 2019 Diseases and Injuries Collaborators, 2020; Arzanova and Mayrovitz, 2022) and is the first cause of death from malignant tumors in women in the world (Smolarz et al., 2022). Breast cancer incidence rates have increased over the last four decades (2010–2019, 0.5% increase per year), largely driven by localized stage and hormone receptor-positive disease (Giaquinto et al., 2022). The most common and widely accepted classification of breast cancer is from an immunohistochemical perspective, based on the expression of estrogen receptor (ER), progesterone receptor (PR), and overexpression of human epidermal growth factor receptor 2 (HER2), and/or amplification of *ERBB2* gene. In this context, there are four molecular subtypes of breast cancer: 1) luminal A (ER and/or PR positive and HER2/neu negative), 2) luminal B (ER and/or PR positive and HER2/neu positive), 3) HER2-positive (ER and PR negative and HER2/neu positive), and 4) triple-negative (ER, PR, and HER2/neu negative) (Patel et al., 2020; Doğan et al., 2023).

The human epidermal growth factor receptor 2 (HER2) is a tyrosine kinase receptor critically involved in the carcinogenesis of the mammary gland (Moasser, 2007). Approximately 20% of - BC cases are HER2 positive (Patel et al., 2020). The study of HER2 oncogenic role and the development of drugs targeting HER2 have revolutionized breast oncology. In the context of HER2-positive early breast cancer (eBC), trastuzumab has emerged as the pivotal cornerstone in the therapeutic landscape. According to seminal studies evaluating adjuvant treatment of HER2+ eBC, the addition of trastuzumab to standard adjuvant chemotherapy halves the risk of recurrence, with a 10% absolute improvement in disease-free survival (DFS) and a 9% increase in 10-year overall survival (OS) (Slamon et al., 2011; Perez et al., 2014; Cameron et al., 2017). In HER2+ disease, as for other BC subtypes, a neoadjuvant strategy is usually preferred to the adjuvant one (Wuerstlein and Harbeck, 2017), except for small tumors (T < 2 cm), clinically node-negative. Dual HER2-targeting with pertuzumab added to chemotherapy plus trastuzumab as neoadjuvant treatment further increased pathologic complete response (pCR) rate (Schneeweiss et al., 2013; Gianni et al., 2016), and led to pertuzumab approval by both US Food and Drug Administration (FDA) and the European Medicines Agency (EMA). In the adjuvant setting, pertuzumab with trastuzumab (PH) showed a benefit in invasive DFS improvement (0.9%), most driven by the high-risk population with node-positive HER2+ eBC (Piccart et al., 2021). In the metastatic setting, most patients receive frontline dual blockade with PH combined with a taxane, followed by dual blockade maintenance (+/- endocrine treatment in tumors expressing HER) (Cardoso et al., 2020). This regimen has led to an unprecedented OS of 57 months, with more than a third of the patients being alive after 8 years (EMA, 2020; FDA, 2020; Swain et al., 2020; Mateo et al., 2022). The previous studies mentioned used intravenous PH formulation. However, subcutaneous (SC) formulations may offer several advantages compared with intravenous (IV), including shorter treatment times, a reduction in the use of healthcare resources, increased convenience for patients, and greater patient preference. In this setting, two robust clinical trials (FeDeriCa and PHranceSCa studies) demonstrated the efficacy, safety and preferences of pertuzumab and trastuzumab fixed-dose combination for subcutaneous injection (PH FDC SC) for the treatment of HER2-positive BC. The Phase 3 pivotal study FeDeriCa compared the pharmacokinetics, efficacy, and safety of PH FDC SC and IV PH in 500 patients with HER2-positive eBC in the neoadjuvant/adjuvant settings (Im et al., 2021; Tan et al., 2021). The Phase 2 PHranceSCa study (O’Shaughnessy et al., 2021) compared the preferences of patients for the administration route for PH FDC SC or PH IV at two-time points: after trying both methods of administration post-surgery, and after completion of neoadjuvant IV PH and chemotherapy. Patients could then choose SC or IV to continue for up to 18 cycles. The primary analysis showed that most patients preferred PH FDC SC (85.0% overall vs. 13.8% for IV PH; 1.3% had no preference). The two main reasons patients preferred PH FDC SC were spending less time in the clinic (42.2%) and being comfortable during administration (25.9%). Indeed, 86.9% of patients choose to continue their HER2-targeted adjunctive therapy with PH FDC SC over IV PH (13.1%) (O’Shaughnessy et al., 2021).

In the PrefHer study, both patients and healthcare professionals (HCPs) demonstrated a preference for SC trastuzumab over the intravenous IV administration method. Additionally, within this study, a prospective, observational time and motion analysis was conducted to quantitatively assess the time that patients spent in infusion chairs and the active time expended by HCPs in managing the PrefHer process. The study had a similar design to PHranceSCa and has demonstrated reductions in patient chair time and active HCP time in eight countries (De Cock et al., 2016). This time-and-motion evaluation showed that, per treatment session, SC administration via a portable syringe (comparable to a single-use injection device) reduced patient chair time (time between entering and exiting the chair infusion) versus IV infusion averaging 55.2 min (mean range of time savings across countries: 40.3–80.6 min; *p* < 0.0001). Such evidence was able to demonstrate that treatment time can also impact the quality of life (QoL) of these patients as well as the use of health resources.

Based on this data, in 2020, the FDA and EMA first approved the ready-to-use fixed-dose combination of PH for subcutaneous (SC) injection (pertuzumab, trastuzumab, and hyaluronidase-zzxf; PH FDC SC) to treat adult patients with HER2-positive BC that has spread to other parts of the body, and for treatment of adult patients with early HER2-positive BC (EMA, 2020; FDA, 2020).

New cancer therapies receive regulatory approval yearly for biomarker-defined subsets of patients, including HER2-positive patients. However, to avoid health disparities in society, the health systems are challenged to adapt their infrastructure, methodologies, and reimbursement policies to allow broad access to these drugs for patients. Broad and equitable access to treatments will depend on the specific situation in various countries and their health systems, in addition to the specificity of patients or tumors. The affordability of new therapeutic strategies is required to ensure health systems’ sustainability (Mateo et al., 2022). Moreover, such affordability is based on an accurate diagnosis. It is well known that this accuracy is impossible to achieve depending on the healthcare system. Access plans for advanced diagnostics need to be designed in a patient-centric rather than institution-centric manner. Clearly, it does not seem feasible that all healthcare institutions can adopt advanced diagnostic platforms and support teams for data interpretation. This gap is part of the problem of accessing new technologies that will provide better treatments for patients (Mateo et al., 2022).

In light of such evidence, the present review aimed to investigate the administration route preferences’ (SC or IV) among patients and HCPs (doctors, nurses, psychologists and others); and to investigate the healthcare resources utilization (quality and quantity) for each route of administration (SC or IV) for treating the patients with HER2-positive BC.

## 2 Methods

This systematic literature review is registered with the International Prospective Register of Ongoing Systematic Reviews (Systematic review registration – PROSPERO 2023: CRD42023412349).

### 2.1 Literature review

The literature search was conducted using PubMed (MEDLINE), Cochrane Library, Virtual Health Library (VHL), Scientific Electronic Library Online (SciELO), and Latin American and Caribbean Health Sciences Literature (LILACS) databases based on the Preferred Reporting Items for Systematic Reviews and Meta-Analysis (PRISMA) statement (Page et al., 2021). The reviews were performed independently by two authors (RBG and JBB) in a blinded fashion way using the Rayyan online platform (Ouzzani et al., 2016). Any discrepancies detected after unblinding were resolved by consensus between RBG, JBB, and TCM.

Our search focused on randomized clinical trials, observational studies, and systematic literature reviews that assessed: 1) patients’ and HCPs’ preferences, perceptions, and satisfaction with SC and IV administration route; and 2) healthcare resources utilization (quality and quantity) for treating the patients with SC and IV administration route. The target population included patients with early or metastatic HER2-positive BC (Supplementary Tables S1, S2).

### 2.2 Search strategy and selection criteria

We searched databases from the first publication until 30 January 2023. The search strategy followed Boolean terms for two categories of focus: 1) patients and HCP preferences, perceptions, and satisfaction; and 2) healthcare resource utilization. For each category, we had a search strategy (Supplementary Tables S3, S4).

Relevant publications from the listed references of the included articles, as well as from other systematic reviews and meta-analyses, were also assessed for eligibility. References were complemented by research on works registered on clinicaltrials.gov.

### 2.3 Eligibility criteria

We considered as inclusion criteria: 1) articles reporting original data; 2) human research; 3) studies with patients with early or metastatic HER2-positive BC; 4) manuscripts written in English, Spanish, French, German or Portuguese; 5) randomized clinical trials, observational studies, and systematic literature review; 6) adult patients aged equal or greater than 18 years old; 7) comparison of the outcomes between SC and IV administration route; and 8) present the outcomes related to the use of trastuzumab or the combination of PH. Regarding exclusion criteria, we considered: (1) book chapters, conference abstracts, case reports, case series, letters, comments, interviews, and narrative reviews; (5) children and adolescents; and (6) overlapped data (in this case, we included the latest published data).

### 2.4 Data extraction

The following variables were extracted according to a structured checklist previously prepared by the authors: 1) metadata (authorship, publication year, study design and country); 2) patients characteristics (sample size and diagnosed disease); 3) characteristics of the intervention (therapy and regimen); 4) measures used to access the outcome of interest; and 5) the outcomes of interest: patients and HCP preferences, resources used/consumed, and cost-savings.

### 2.5 Quality assessment

To evaluate the quality of the evidence, we used the corresponding tool for each study design: 1) Randomized clinical trials - Risk of Bias for randomized trials version 2.0 (RoB 2.0) (Sterne et al., 2019); 2) Observational studies - Risk Of Bias In Non-randomized Studies - of Interventions (ROBINS-I) (Sterne et al., 2016); and 3) Economic model studies - Consolidated Health Economic Evaluation Reporting Standards (CHEERS) (Husereau et al., 2013).

## 3 Results

### 3.1 Overview

**FIGURE 1:**
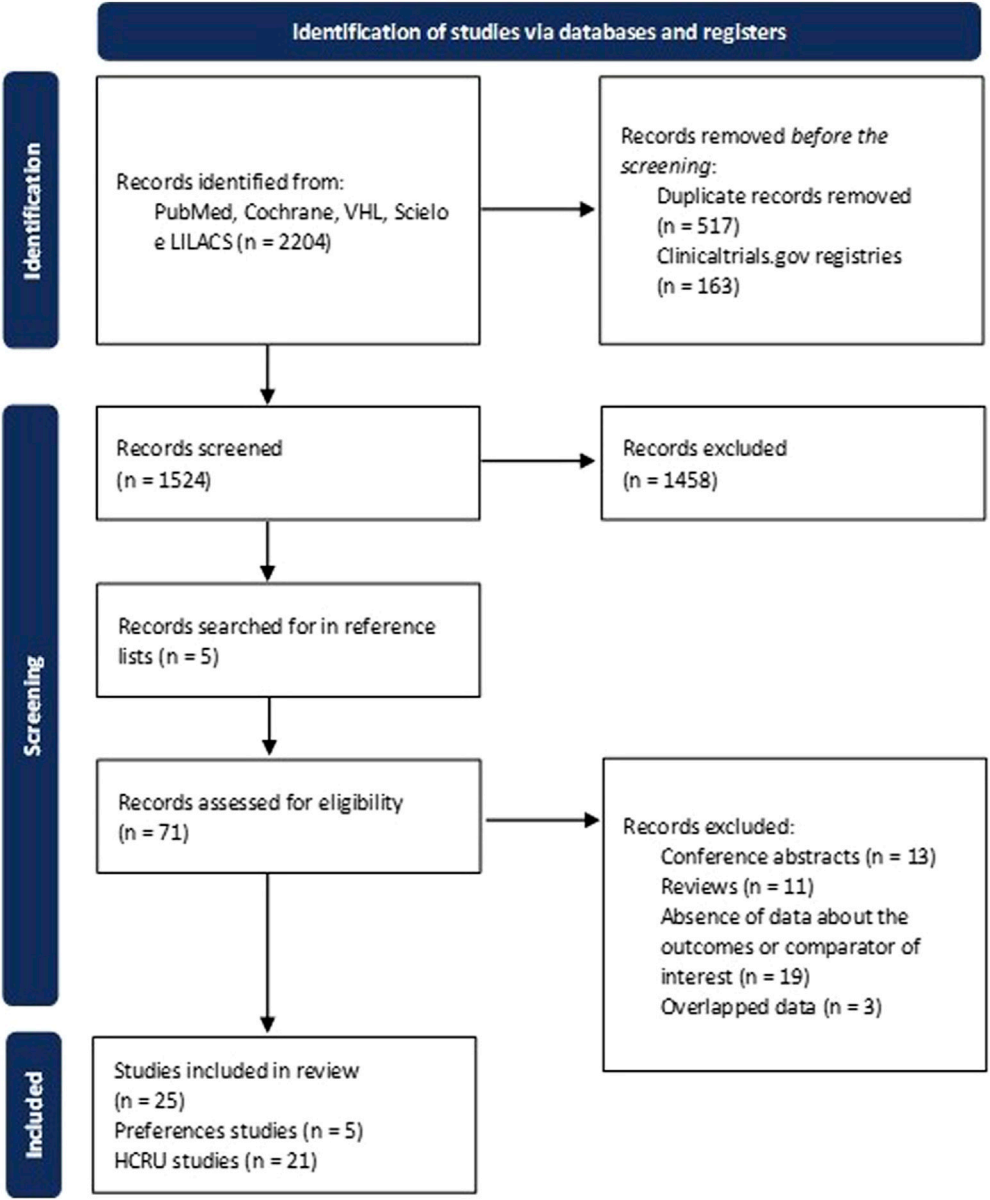
Selection of the studies flowchart. HCRU, healthcare resource utilization.

Our systematic review yielded 1,524 studies after duplicates were removed. In a preliminary eligibility evaluation, we excluded 1,458 articles (Figure 1). In a more detailed subsequent selection phase, we excluded 46 articles for the following reasons: incorrect study design (abstracts and reviews) (n = 24); absence of data of the outcome or comparator of the interest (n = 19), and overlapped data (n = 3), meaning that we used the latest published data (Supplementary Table S5). In the end, 25 studies complied with our criteria and were included for the analyses: 5 studies for patients and HCP preferences and 21 studies for the outcomes of healthcare resource utilization. Notably, the study by O’Shaughnessy et al. (2021) (O’Shaughnessy et al., 2021), was included in both categories due to its comprehensive data on preferences and HRCU.

Table 1 and Supplementary Table S7 summarizes the characteristics of all included studies (preferences and healthcare resource utilization). In total, 25 publications were identified that described the preferences and healthcare resource utilization in terms time/resource use and/or costs associated with the comparison of SC versus IV administration for the treatment of HER2-positive BC.

**TABLE 1:** Characteristics of the included studies regarding preferences for each administration route.

Author, year	Study design	Diagnose	Therapy	Regimen	Preference measure	Sample size Sc	Sample size iv	Median age (yrs)	Main findings
Pivot et al. (2014)	Open-label, randomized study [PrefHer (NCT01401166)] - data from two cohorts	Early HER2-overexpressing breast cancer	Trastuzumab	-Neoadjuvant chemotherapy followed by SC trastuzumab (600 mg) for 18 cycles followed by IV (standard dosing) compared with the reverse; -Cohort 1: SC by injection device; -Cohort 2: SC by handheld syringe	Patients: telephone interviews and self-administered satisfaction questionnaire; HCP: satisfaction question (‘All things considered with which method of administration were you most satisfied?’) and perceived time savings	SC → IV: 235	IV → SC: 232	52–53	Patients Preferences: 88.9% preferred SC (415/467, 95% CI 85.7–91.6; *P*< 0.0001), 9.6% (45/467, 95% CI 7.1–12.7) preferred IV, and 1.5% (7/467, 95% CI 0.6–3.1) had no preference Main reasons: time-saving and less pain/discomfort/side effects HCPs Preferences: 77.0% preferred SC (181/235, 95% CI 71.1–82.2), 3.0% (7/235, 95% CI 1.2–6.0) preferred IV, and 20.0% (47/235, 95% CI, 15.1–25.7) had no preference
Reinisch et al. (2022)	Substudy of the phase III multicenter, randomized trial [GAIN-2 (NCT01690702)]	HER2-positive breast cancer [(neo)adjuvant chemotherapy and surgery]	Trastuzumab	-SC: 600 mg fixed dose; -IV: loading dose of 8 mg/kg and subsequent doses of 6 mg/kg 18 triweekly dosing cycles (1 year of treatment/a full treatment cycle)	Patients: validated, study-specific patient interview (PINT) questionnaires before randomization (PINT1) and after the end of cycle 8 of SC trastuzumab (PINT2)	SC thigh: 110 SC AW: 109	IV: 219	50	Patients Preferences: 83.5% (152/182) preferred SC over previous IV applications or had no preference None of the SC sites of injection were preferred over the other (thigh: N = 93 (80.6% [95% CI 72.6–88.7]); AW: N = 89 (86.5% [95% CI 79.4, 93.6]), *p* = 0.322; odds ratio (OR) 1.54 [95% CI 0.69–3.42], *p* = 0.288)
O’Shaughnessy et al. (2021)	Randomized, open-label, international, multicenter, crossover, phase II study conducted at 39 sites in 16 countries [PHranceSCa (NCT03674112)]	Early HER2-overexpressing breast cancer	PH FDC SC P+H IV	Loading doses: -IV: P IV 840 mg; H IV 8 mg/kg; -SC: PH FDC SC 1200 mg P/600 mg H in 15 mL Maintenance doses: -IV: P IV 420 mg; H IV 6 mg/kg; -SC: PH FDC SC 600 mg P/600 mg H in 10 mL	Patients: modified intention-to-treat (mITT) population - the proportion of patients who preferred PH FDC SC based on the question: “All things considered, which method of administration did you prefer?” [Patient Preference Questionnaire (PPQ)]	PH FDC SC → P + H IV: 80	P + H IV → PH FDC SC:80	47	Patients Preferences: -PH FDC SC: 85.0% (136/160) - “very/fairly strong” preference: 92.6% [the most common reasons were “requires less time in the clinic” and “feels more comfortable during administration”] -P + H IV: 13.8% (22/160) - “very/fairly strong” preference: 63.6% [the most common reasons were “feels more comfortable during administration” and “lower level of injection site pain”] Patients perceptions: -“(very) satisfied” - PH FDC SC: 88.1%; P + H IV: 67.5%; -“not at all” restricted - PH FDC SC: 71.3%; P + H IV: 34.4%; -“gained a lot of time” or “gained some time” - PH FDC SC: 60.6%; P + H IV: 4.4% HCPs Preferences: −86.9% (139/160) chose to continue with PH FDC SC after completing the crossover (arm A: 88.8% [71/80]; arm B: 85.0% [68/80])
Pivot et al. (2017)	Open label, randomized, multicenter, phase III study [MetaspHer (NCT01810393)]	Metastatic HER2-overexpressing breast cancer	Trastuzumab	-SC: 3 cycles of 600-mg fixed-dose; IV: 6 mg/kg	Patients: Patient Preference Questionnaire (PPQ); HCP: Satisfaction questionnaire	SC → IV: 47	IV → SC: 45	57.8–59.5	Patients Preferences: -SC: 85.9% (79/92; 95% CI: 78.8%–96.8%; *p* < 0.001); IV: 14.1% (13/92; 95% CI: 7.0%–21.3%); -Among patients without preference at baseline (52/89 available data) SC was the preferred administration route - SC: 88.5% (46/52; 95% CI: 79.8%–97.2%) HCPs Preferences: -SC: 63.6% were satisfied (56/88 available data; 95% CI: 53.6e73.7%)
Ciruelos et al. (2020)	Phase III, open-label, multicenter study [ChangHER (NCT01875367)]	Metastatic HER2-overexpressing breast cancer	Trastuzumab	SC: 600 mg every 3 weeks for 4 cycles -arm A (2 cycles with vial followed by 2 cycles with SID); -arm B (reverse sequence) Before starting SC, patients received an additional IV cycle	Questionnaire (the study did not report the name of the instrument)	IV→ vial → SID: 85	IV → SID → vial: 81	58–63	Patients Preference: -SC: 86.2%; IV: 6.9%; had no preference: 6.9% -arm A (vial to SID) - SC: 86.8% (95% CI 77.1–93.5); IV: 7.9% (95% CI 3.0–16.4); had no preference: 5.3% (95% CI 1.5–12.9); -arm B (SID to vial) - SC: 85.5% (95% CI 76.1–92.3); IV: 6.0% (95% CI 2.0–13.5); had no preference: 8.4% (95% CI 3.5–16.6) HCPs Preferences (nurses, medical oncologists, and others): -SC: 87.2% (95% CI 72.6–95.7); no difference: 10.3% (95% CI 2.9–24.2); failed to respond: 2.6% -The most important factors associated with the SC preference: “fewer resources required for preparation” (100%); “time saver” (97.4%); “more convenient for patients” (94.9%); and “less painful for patients” (76.9%)

HER2, human epidermal growth factor receptor 2; SC, subcutaneous; IV: intravenous; CI, confidence interval; P, Pertuzumab (Perjeta); H, trastuzumab (Herceptin); P + H IV, intravenous pertuzumab plus trastuzumab; PH FDC SC, fixed-dose combination of Perjeta and Herceptin for subcutaneous injection; HCP, healthcare professionals; AW, abdominal wall; SID, single injection device.

Concerning the variable of healthcare resource utilization (Table 1), two publications were related to PrefHer, a multinational study conducted in eight countries (Canada, France, Switzerland, Denmark, Italy, Russia, Spain, and Turkey) (Jackisch et al., 2015; De Cock et al., 2016), 16 publications reported data from European countries (Burcombe et al., 2013; Jackisch et al., 2015; Lieutenant et al., 2015; Lazaro Cebas et al., 2017; De Cock et al., 2016; Olofsson et al., 2016; Farolfi et al., 2017; Lopez-Vivanco et al., 2017; Olsen et al., 2017; Tjalma et al., 2018; Hedayati et al., 2019; Mitchell and Morrissey, 2019; O’Brien et al., 2019; Altini et al., 2020; O’Shaughnessy et al., 2021; Simoens et al., 2021). The remaining studies (n = 5) were from Costa Rica (Cordero et al., 2019), Brazil (Kashiura et al., 2019), Chile (Rojas et al., 2020), Saudi Arabia (Elsamany et al., 2020), and New Zeland (North et al., 2015). Regarding the study design, nine studies were focused in reporting a health economic model of cost-effectiveness (Tjalma et al., 2018; Hedayati et al., 2019), budget impact (Kashiura et al., 2019; Elsamany et al., 2020), cost-minimization (North et al., 2015; Cordero et al., 2019; Rojas et al., 2020), and micro-costing (Lopez-Vivanco et al., 2017; O’Brien et al., 2019). Of those, three studies had data based on the PrefHer Trial (NCT01401166) (Lopez-Vivanco et al., 2017), SafeHer Trial (NCT01566721) (North et al., 2015), and HANNAH Trial (NCT00950300) (Kashiura et al., 2019). The remaining 11 studies were designed as observational (prospective cross-sectional and cohorts) and, four of them were based on the PrefHer Trial (NCT01401166) (Burcombe et al., 2013; Jackisch et al., 2015; De Cock et al., 2016; Farolfi et al., 2017). Finally, one was the randomized, open-label, international, multicenter, crossover, phase II PHranceSCa Trial (NCT03674112) (O’Shaughnessy et al., 2021). Eleven studies specified the stage of the diagnose of the breast cancer, being eight with individuals diagnosed with HER2-positive eBC (Burcombe et al., 2013; North et al., 2015; De Cock et al., 2016; Farolfi et al., 2017; Lopez-Vivanco et al., 2017; Mitchell and Morrissey, 2019; Rojas et al., 2020; O’Shaughnessy et al., 2021) and three HER2-positive early or metastatic BC (Olofsson et al., 2016; Tjalma et al., 2018; Kashiura et al., 2019). Regarding the therapy, only one study demonstrated data regarding the combination of PH (O’Shaughnessy et al., 2021), while the others were conducted with trastuzumab. Seventeen studies reported data regarding resource utilization in terms of patient and HCP time spent to conduct the administration of the medication, while 18 studies reported data regarding the cost related to the treatment (per cycle or full-cycle treatment).

Studies related to the variable of patients’ and HCPs’ preferences (Supplementary Table S7), each of the five included studies were from a different randomized clinical trial: PrefHer (NCT01401166) (Pivot et al., 2014), GAIN-2 (NCT01690702) (Reinisch et al., 2022), PHranceSCa Trial (NCT03674112) (O’Shaughnessy et al., 2021), MetaspHer (NCT01810393) (Pivot et al., 2017), and ChangHER (NCT01875367) (Ciruelos et al., 2020). Two studies were conducted with individuals diagnosed with HER2-positive metastatic BC (Pivot et al., 2017; Ciruelos et al., 2020) and the remaining three were with individuals diagnosed with HER2-positive eBC (Pivot et al., 2014; O’Shaughnessy et al., 2021; Reinisch et al., 2022). As mentioned previously, only one study was conducted with the combination of PH (O’Shaughnessy et al., 2021), while the others were conducted with trastuzumab only. Most of the included studies presented data regarding patients’ and HCPs’ preference; one study from Reinisch et al. (Reinisch et al., 2022) (substudy of the phase III trial GAIN-2 [NCT01690702]) reported data of patients’ preference only.

### 3.2 Main results for patients and healthcare professional preferences

**FIGURE 2:**
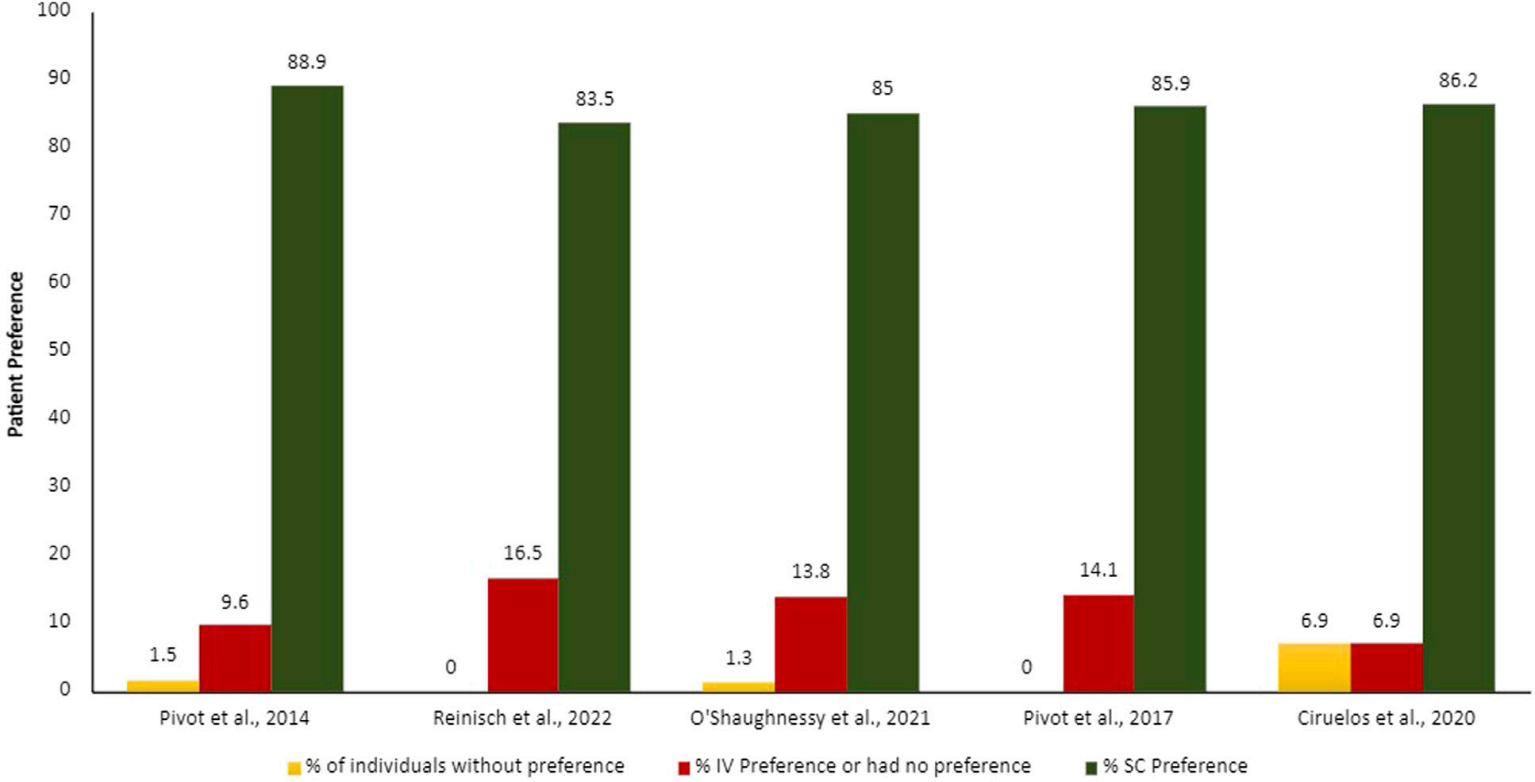
Proportion of patients’ preferences according to each administration route. IV, intravenous; SC, subcutaneous.

**FIGURE 3:**
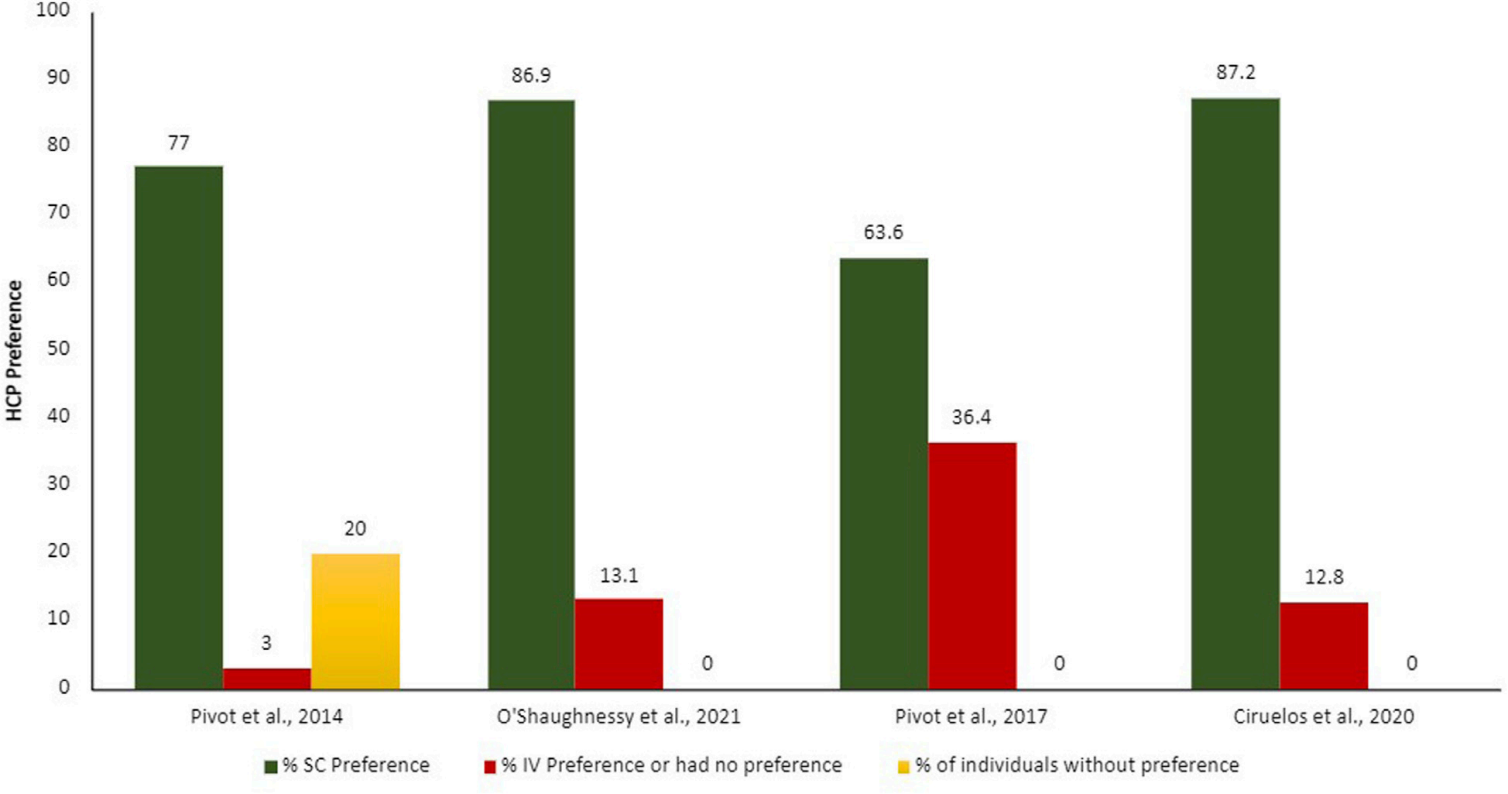
Proportion of HCPs preferences according to each administration route. IV, intravenous; SC, subcutaneous.

Summarized results from patients and HCP preferences can be found in Figures 2, 3.

The patients and HCPs’ preferences were evaluated though different measures, for example, semi-structure interviews with open questions, validated study-specific patient interview, validated preference questionnaire (Patient Preference Questionnaire [PPQ]), and satisfaction questionnaires. The output of these evaluations was demonstrated in proportion of patients and HCPs who prefer each administration route. Overall, more than 75% of the patients and HCPs preferred the SC administration route over the IV.

The main reasons demonstrated by the studies on why patients prefer SC administration route include time-savings and less pain, discomfort, and side effects (Pivot et al., 2014; O’Shaughnessy et al., 2021); HCPs agreed that SC administration route is time-savings, more convenient and less painful for patients, in addition to requiring fewer resources for preparation (Ciruelos et al., 2020). Promoting benefit in the HCPs workload, reducing drug waste, enabling shorter infusion times and observation of attack and maintenance drug doses, generating a significant reduction in patient chair time.

### 3.3 Main results for healthcare resource utilization

The summarized results of the healthcare resource utilization can be found in Tables 2, 3.

**TABLE 2:** Summarized results regarding healthcare resource utilization in terms of time spent for patients and HCP.

Authors, year	Healthcare resource utilization (time)^a^	Relation SC:IV
Cordero et al. (2019)	Administration time	1:3
Mitchell and Morrissey (2019)	Chair-time per session	1:4
Olofsson et al. (2016)	Time off from work	1:3
	Time for the accompanying kin	1:1
Lieutenant et al. (2015)	Administration time (Loading doses Administration time (Maintenance doses)	1:4 1:2
	Transit time	1:18 to 1:6
	Manufacturing time	1:3
O’Brien et al. (2019)	Treatment room time	1:4
Lopez-Vivanco et al. (2017)	HCP time	1:2
	Chair-time	1:5
	Treatment room time	1:4
	Hospital time	1:2
North et al. (2015)	HCP time	1:2
	Chair-time	1:5
Burcombe et al. (2013)	HCP time	1:4
	Time at the unit of care	1:3
	Chair-time	1:4
Tjalma et al. (2018)	HCP time	1:6
	Hospital time	1:3
	Chair-time	1:13
Altini et al. (2020)	Administration time	1:2
	Chair-time	1:2
Farolfi et al. (2017)	Preparation time	1:3
	Administration time	1:9
Hedayati et al. (2019)	Administration time (1st session) Administration time (Subsequent sessions)	1:9 1:3
Jackisch et al. (2015)	HCP time	1:2
	Chair-time	1:3
O’Shaughnessy et al. (2021)	Chair-time	1:4 to 1:6
	Administration time	1:9 to 1:19

^a^ Time was measured according to the study methodology (hours or minutes).

**TABLE 3:** Summarized results regarding healthcare resource utilization in terms of treatment costs.

Authors, year	Country	Healthcare resource utilization (costs)^a^	Relation SC:IV
Cordero et al. (2019)	Costa Rica	Cost per application	1:6
Mitchell and Morrissey (2019)	United Kingdom	Total cost	1:3
Olofsson et al. (2016)	Sweden	Societal treatment costs (First-time treatment occasion) Societal treatment costs (Subsequent treatment occasions)	1:1 1:1
O’Brien et al. (2019)	Ireland	Costs of Consumables (Per treatment cycle) Costs of Consumables (For a complete 17-cycle treatment) HCP Costs (Preparation and administration - Per treatment cycle) HCP Costs (Preparation and administration - For a complete 17-cycle treatment) Drug Costs (17-cycle treatment) Indirect Costs (Lost productivity for 17-cycle treatment per patient)	1:2 1:2 1:5 1:5 1:1 1:3
Lopez-Vivanco et al. (2017)	Spain	Costs of Consumables (Per treatment cycle) Costs of Consumables (For a complete 18-cycle treatment) HCP Costs (Preparation and administration - Per treatment cycle) HCP Costs (Preparation and administration - For a complete 18-cycle treatment) Drug costs (18-cycle treatment) Indirect costs (lost productivity - By patient room time) Indirect costs (lost productivity - By hospital time	1:4 1:4 1:2 1:2 1:1 1:4 1:2
Burcombe et al. (2013)	United Kingdom	Costs/patient episode (administration and preparation)	1:4
Tjalma et al. (2018)	Belgium	Total cost HCP time/patient episode Cost of consumables	1:20 1:5 1:8
Altini et al. (2020)	Italy	Total cost	1:1
Elsamany et al. (2020)	Saudi Arabia	Costs to prepare and administer the drugs formulations over 3 years Total annual costs (drug and non-drug costs) - 1st scenario Total annual costs (drug and non-drug costs) - 2nd scenario Indirect costs (lost productivity)	1:12 1:2 1:2 1:25
Lazaro Cebas et al. (2017)	Spain	Total cost	1:1
Farolfi et al. (2017)	Italy	Total cost of the drugs Direct cost/patient Outpatient clinic costs/patient Direct + Indirect costs (costs/patient)	1:1 1:1 1:9 1:1
Rojas et al. (2020)	Chile	HCP Costs (Preparation - Per treatment cycle) HCP Costs (Preparation - For a complete 18-cycle treatment) HCP Costs (Administration - For a complete 18-cycle treatment) Adverse drug reaction (ADR) treatment costs Non-medical costs Total cost	1:1 1:1 1:2 1:1 1:1 1:1

^a^ Currency was standardized in United States Dollars (USD) on 27 March 2023.

### 3.4 Healthcare professionals and patients’ time

In terms of the variable’s definition in the included studies, HCP (e.g., pharmacists, nurses, nursing assistants, medical staff, etc.) time includes drug preparation and administration times. Chair time refers to the period that the patients spent in the unit of care to receive the drugs (entry and exit from the infusion chair), also referred to as treatment room time, time at the unit, and hospital time. Additionally, as a patient variable, some studies report data regarding the burden of the treatment in the patients and caregivers’ life, referring to time off from work and transit.

Specifically for HCP time, the studies reported that IV administration time can be two to 19 times longer than SC administration time (including loading and maintenance doses), while the preparation time for IV can be three times longer than SC. Regarding the overall HCP time (including administration and preparation time), IV administration time can be two to six times longer than SC administration time.

Regarding the patients’ time spent to receive the drugs, the studies reported significant time-savings with the SC administration route. Intravenous administration makes the patient remain in the care unit for two to 13 times longer compared to SC, which also prolongs work absences by three times.

Overall, the SC administration route saves more than 40% of HCP and patients time compared to IV.

### 3.5 Costs related to SC compared to IV administration route

The reported costs by the included studies were based on data from time-and-motion, in which the time for specific procedures was directly measured. Other studies reported direct costs, expressed by the resource used, for example, drugs, consumables, healthcare personnel, catheter, possible waste of the drug, structural costs, and adverse events; as well as indirect costs, expressed by the burden that this procedure imposes to patients and HCPs, like societal costs and loss of productivity. Those costs were also extrapolated for one to 5 years of treatment.

The studies from Simoens et al. (Simoens et al., 2021), Lieutenant et al. (Lieutenant et al., 2015), North et al. (North et al., 2015), Olsen et al. (Olsen et al., 2017), did not present the exact cost (in terms of values) comparison between IV and SC, but demonstrated the significant cost-saving of using the SC administration route. Specifically, the case study of Simoens et al. (Simoens et al., 2021) in Belgium healthcare center found that IV treatment was less expensive than SC for patients weighing up to 75 kg. This phenomenon occurred because the authors considered data from biosimilars to conduct the study. Kashiura et al. (Kashiura et al., 2019) demonstrated the budget impact of incorporating the SC administration route in Brazilian private healthcare system for a period of 5 years and reported a significant cost-saving compared to IV administration route (cost-savings of up to USD 176,859,259.46 for HER-2 positive eBC and up to USD 6,307,656.20 for HER-2 positive metastatic breast cancer). Hedayati et al. (Hedayati et al., 2019) demonstrated that SC administration can save USD 650,710.94 over 1 year, avoiding surgery to implant catheters (69% of cost-saving), and saving time for drug preparation (28% of cost-saving) and consumables (3% of cost-saving) involved in the procedure.

Regarding direct costs with consumables, the studies reported that the IV administration route cost two to four times more than SC; the costs of health professionals, which include the preparation and administration of the medication, are one to eight times higher in the IV administration route in comparison to SC per cycle and for full cycles (17–18 cycles); if we extrapolate these data to 3 years of treatment, these costs could be 12 times higher with the IV administration route; indirect costs vary from one to 25 times higher when using the IV administration route; structural costs are also higher with the IV administration route–which is nine times higher than with SC. Interestingly, total costs and drug and adverse event costs did not differ when comparing IV to SC administration route.

### 3.6 Quality assessment

In the Supplementary Figures S1, S2 and Supplementary Table S6, we demonstrated the results regarding the quality appraisal of the included studies. Overall, most of the observational studies presented low to moderate risk of bias. Only three studies demonstrated serious risk of bias due to: deviations from intended interventions and missing data (Altini et al., 2020); measurement of the outcomes (Mitchell and Morrissey, 2019); and classification of the interventions (Burcombe et al., 2013). Regarding the risk of bias of randomized controlled trials, we found that more than half of the included studies presented low to some concerns. Only two studies demonstrated high methodological risk of bias due to: randomization process (Ciruelos et al., 2020; O’Shaughnessy et al., 2021) and selection of the reported result (Ciruelos et al., 2020). The economic studies were evaluated by the CHEERS checklist. Bias was considered when the study did not report some of the mandatory item for conducting an economic study design. Overall, topics not reported by some studies were: 1) discount rate and its reason for including 2) currency, price date and conversion; 3) characteristics of heterogeneity and uncertainty; 4) specific parameters; 5) effect of uncertainty; and 6) conflict of interests.

## 4 Discussion

This systematic literature review focused on the benefits of biologic administration routes for the treatment of early-stage or metastatic HER-2-positive BC, regardless of the drugs administered. These benefits were evaluated through preferences reported by HCPs and patients, time spent performing this task, and cost savings. According to our study, the HCPs and patients prefer the SC administration route. Furthermore, and consistent with these findings, the SC method of administration substantially reduces the time spent by HCPs on administration and preparation, as well as patient chair time in the healthcare facility. The advantages of SC therapy are understood to include shorter treatment time, reduced use of healthcare resources, lower costs (both direct and indirect costs), greater patient convenience, and greater preference for patients and HCPs when compared to IV therapy (Pivot et al., 2013; Wynne et al., 2013; Pivot et al., 2014; De Cock et al., 2016). Another possible advantage of the SC administration route is that patients do not need to go to an infusion room; treatment can be administered by trained nurses outside the hospital setting (O’Shaughnessy et al., 2021).

In light of these considerations, the SC administration route emerges as an enticing solution, further augmented by its capacity to offer the convenience of home delivery. Administration at home reduces the risk of exposure to nosocomial infections. It is expected that, with this alternative, the QoL of patients will improve, in addition to making life easier for those who live far from a hospital or have difficulties in commuting and parking close to the hospital. This can contribute to a lower financial, family, and friends burden (Jonaitis et al., 2021). However, some countries, like Brazil, may have specific legislation that restricts the use of cancer therapies to certified units.

SC delivery systems are designed with smaller needle sizes, which can decrease pain during administration. It has been proven effective, safe, well tolerated, and generally preferred by patients and HCPs because it is less time-consuming, requires less effort and time absent from work, reduces the loss of productivity and leisure time associated with patients attending the hospital, and minimizes the discomfort associated with IV infusions. The SC route of administration, interestingly, results in the reduction of health costs related to drug administration and the use of resources and is cost saving from the societal perspective (Jonaitis et al., 2021). Another possible benefit is that central venous access devices can be removed sooner, reducing the risk of morbidity (O’Shaughnessy et al., 2021). These benefits are particularly noticeable in the context of the public health system, where human resources are limited.

It is important to highlight that the decision of the treatment and route of administration, should be shared with patients. In the decision-making process, patients need to understand the relative time-related burden associated with different treatment options. Although values and preferences will vary across individuals, most patients want to minimize time toxicity. Most clinical trials do not report measures of time toxicity. This data could be used to guide patients, who might have different priorities (O’Shaughnessy et al., 2021). With respect to transition costs from IV to SC administration, the SC administration route may offer payers distinct cost advantages. Compared to IV infusions, many SC-administered drugs (e.g., rituximab and belimumab) offer direct cost savings as they do not require premedication (Heald et al., 2021). As an example of this direct cost reduction, the assessment of the budgetary impact (forecasted budget impact at 1, 2 and 3 years) of introducing rituximab SC in cancer patients in US health plans showed that changing the route of administration from IV to SC reduced total pharmacy and administration costs in the year of highest conversion rate by $223,000 (translating to a per-member-per-month [PMPM] decrease of $0.02) (Tetteh and Morris, 2014; Hansen et al., 2018). Similar findings with oncology biologics have been reported across countries despite differences in healthcare systems and payer types (Heald et al., 2021). A Brazilian study demonstrated that incorporating the SC administration route into the private system resulted in a significantly lower budgetary impact when compared to the IV administration route (Kashiura et al., 2019). It is important to mention that this study was conducted with the reference drug and the magnitude of savings can vary according to the type of drug (biosimilar or reference) and the context of the health system (public or private). Additionally, one potential challenge with SC administration is the use of fixed doses, which may not account for interpatient variability in body weight and surface area. This could lead to insufficient dosing in larger patients or excessive dosing in smaller patients. However, studies have shown that the fixed-dose regimen of PH FDC SC is generally well-tolerated and effective across a range of patient demographics, although careful monitoring and individual adjustments may be necessary in certain cases to optimize therapeutic outcomes (Kolberg et al., 2021).

Examining indirect costs alongside direct costs is another important consideration for some payers when comparing IV versus SC administration. A cost analysis showed that SC administration costs were 50% lower compared to the IV route, with most patients administering their own SC medications. Other indirect benefits of this administration route include shorter waiting time at the infusion unit, reduced risk of infections or other diseases (especially for patients with breast cancer who are often immunosuppressed), and reduction of direct costs of the patient (travel, occupational break). For biologics cases (IV versus SC), in direct/indirect cost analysis, excluding drug acquisition costs, SC administration appears to be the most cost-effective option for many patients (Heald et al., 2021).

In line with this information, studies have demonstrated that SC administration of biotherapeutics is a relevant alternative to IV administration in diverse disease scenarios, including inflammatory bowel disease, non-Hodgkin’s lymphoma, rheumatoid arthritis, primary immunodeficiency, multiple sclerosis, etc. (Bittner et al., 2018). With the alternative of SC administration, a significant benefit is expected for patients receiving monotherapy of a biologic in the maintenance/adjuvant setting or in combination with oral chemotherapy, as there will be a reduction in the time required for frequent hospital visits. For complex dosing regimens, such as fixed-dose combinations (two or more active molecules co-formulated in the same formulation) or ready-to-use devices that deliver two or more biotherapeutics per half hour from a single SC injection, the use of SC administration can further simplify medication administration (Bittner et al., 2018). Additionally, the SC administration route is as well-tolerated as the IV route, with comparable safety profiles. SC administration often results in localized injection site reactions, such as mild pain, redness, and swelling, which are generally manageable. In contrast, IV administration is associated with a higher incidence of systemic infusion-related reactions, including fever, chills, nausea, headaches, and potential cardiac toxicity. This data indicates that SC administration, with its lower incidence of systemic adverse effects and greater patient convenience, may be a preferable option for many patients undergoing treatment for HER2-positive breast cancer (Pivot et al., 2014; O’Shaughnessy et al., 2021).

Furthermore, the humanistic impact of SC and IV formulations of oncology therapies showed that patients have a clear preference for SC administration and report better health-related QoL (Anderson et al., 2019; Epstein, 2021). Corroborating this fact, patients reported “time savings” as the main reason for preferring SC (Gianni et al., 2010; McCloskey et al., 2023), in addition to being more comfortable, well-tolerated, safe, and less painful. HCPs were also more satisfied with SC as they perceived better clinical management and an efficient method (Marty et al., 2005; Pivot et al., 2014; Gianni et al., 2016).

Patients and HCPs are convinced that the SC administration route is more suitable for younger and employed patients, while the IV route is more suitable for older patients, especially those who refuse to inject themselves and feel safer when receiving therapy in a hospital setting (Jonaitis et al., 2021). The key drivers for switching from IV to SC administration of biologics include medical considerations (disease amelioration/stabilization, facility decongestion, patient involvement in treatment), patient considerations (preference for a more comfortable and easy-to-administer formula, self -administration, a more flexible schedule, limited reliance on medical facilities and personnel) and administrative considerations involving costs and, in some countries, insurance reimbursement (Jonaitis et al., 2021).

Nonetheless, it is important to interpret the data presented in this systematic literature review with a mindful consideration of certain limitations. Firstly, it is important to notice that the efficacy and safety profiles of the medication administered by SC and IV were assumed to be comparable (Kolberg et al., 2021). Secondly, there was some variation in times reported for IV and SC preparation and administration, which may reflect a heterogeneity concerning the methods of measuring the data and its results, for example, the time estimate methodologies, definitions of time periods, and clinical practice/hospital set up between the different participating centers. Based on this premise, it is highly essential to standardize the data measurement methodology and create uniform parameters to adequately support decision-making. Pharmacoeconomic consideration is a point of interest, but they are highly dependent on the model of reimbursement and valorization of IV and/or SC administrations and it could not be translated from one country to another. Independently, of the cost and payment considerations, the SC administration route has demonstrated benefits in terms of time and resource saving, in addition, to being preferred by the HCPs and patients (Pivot et al., 2017).

In conclusion, this systematic literature review highlighted a consistent trend in favor of SC administration across all publications, related to patients and HCP preferences. Combined data, has shown that SC administration route benefits both patients and healthcare systems (Pivot et al., 2014). These data provide supporting evidence for a practice change regarding the route of administration of the anti-HER2 therapy setting either in the adjuvant or in the metastatic setting (Pivot et al., 2017).
