# Supplementary material for: The burden of systemic therapy administration route in treating HER2-positive breast cancer (for patients, healthcare professionals, and healthcare system): a systematic literature review
Source: Front Pharmacol. 2024 Aug 19;15:1338546. doi: 10.3389/fphar.2024.1338546 (PMC11366779; doi:10.3389/fphar.2024.1338546)
Supplement: Supplementary file 1 [file DataSheet1.docx]

**Supplementary Material**

**Research questions demonstrated by the acronym PICO (Population, Intervention, Comparator, and Outcomes)**

*Preference Outcomes*

**Research question:** Which route of administration (SC or IV) of medication do patients with HER2-positive breast cancer and the health team (doctors, nurses, psychologists, etc.) who treat them prefer?

**Table S1.** Research question variables for preference outcomes

| **Variables** | **Components of variables** |
| --- | --- |
| Population | Adult women with HER2-positive breast cancer (≥ 18 years)  Health team (doctors, nurses, psychologists, social workers, etc.) |
| Intervention | SC administration of treatment medication (trastuzumab or trastuzumab + pertuzumab combination) |
| Comparator | IV administration of treatment medication (trastuzumab or trastuzumab + pertuzumab combination) |
| Outcome | Preferences of the patient and health team measured through validated sessions or open interviews (qualitative studies) with the individual |

*Healthcare Resource Utilization Outcomes*

**Research question:** What and how many health resources are used for each route of administration (SC or IV) of the drug in HER2-positive breast cancer patients?

**Table S2.** Research question variables for healthcare resource utilization outcomes

| **Variables** | **Components of variables** |
| --- | --- |
| Population | Adult women with HER2-positive breast cancer (≥ 18 years) |
| Intervention | SC administration of treatment medication (trastuzumab or trastuzumab + pertuzumab combination) |
| Comparator | IV administration of treatment medication (trastuzumab or trastuzumab + pertuzumab combination) |
| Outcome | Use of health resources |

**Search Strategies**

*Preference Outcomes*

**Table S3.** Search Strategy for each included database for preference outcomes

| **Database** | **Search Strategy** |
| --- | --- |
| Cochrane Library | #1 pertuzumab  #2 trastuzumab  #3 (breast cancer):ti,ab,kw  #4 MeSH descriptor: [Breast Neoplasms] explode all trees  #5 (preference):ti,ab,kw  #6 (prefer*):ti,ab,kw  #7 (experience*):ti,ab,kw  #8 (satisfaction):ti,ab,kw  #9 (perspective*):ti,ab,kw  #10 (#1 OR #2) AND (#3 OR #4) AND (#5 OR #6 OR #7 OR #8 OR #9) |
| PubMed - MEDLINE | ((((((((((breast cancer[MeSH Terms]) OR (breast neoplasm[MeSH Terms])) OR (breast neoplasms[MeSH Terms])) OR (HER2-positive[Title/Abstract] AND breast cancer[Title/Abstract])) AND (((((((((drug administration routes[MeSH Terms]) OR (route*[Title/Abstract])) OR (frequenc*[Title/Abstract])) OR (administration[Title/Abstract])) OR (intraveno*[Title/Abstract])) OR (subcutan*[Title/Abstract])) OR (administration, intravenous[MeSH Terms])) OR (Injections, Subcutaneous[MeSH Terms])) OR (Infusions, Subcutaneous[MeSH Terms])))) AND (((((((((((((preference*[Title/Abstract]) OR prefer*[Title/Abstract])) OR (choice behavior[Title/Abstract])) OR (decision making[Title/Abstract])) OR (experience*[Title/Abstract])) OR (expectation*[Title/Abstract])) OR (satisfaction*[Title/Abstract])) OR (perspective*[Title/Abstract])) OR (patient value*[Title/Abstract])) OR (patient-reported outcome*[Title/Abstract])) OR (Patient Preference[MeSH Terms])) OR (patient preferences[MeSH Terms])) OR (Patient Satisfaction[MeSH Terms])) OR (decision making[MeSH Terms])) OR (adher*[Title/Abstract])) OR perception[Title/Abstract])) AND ((((trastuzumab[Title/Abstract]) OR (pertuzumab[Title/Abstract])) OR ((trastuzumab[MeSH Terms]) OR (pertuzumab[Supplementary Concept])))) |
| Virtual Health Library | ti:(((pertuzumab) OR (trastuzumab)) AND (breast cancer) AND ((prefer*) OR (experience*) OR (satisfaction) OR (perspective))) |

*Healthcare Resource Utilization Outcomes*

**Table S4.** Search Strategy for each included database for healthcare resource utilization outcomes

| **Database** | **Search Strategy** |
| --- | --- |
| Cochrane Library | #1 pertuzumab  #2 trastuzumab  #3 (breast cancer):ti,ab,kw  #4 MeSH descriptor: [Breast Neoplasms] explode all trees  #5 MeSH descriptor: [Health Care Rationing] explode all trees  #6 (healthcare resource*):ti,ab,kw  #7 (healthcare utilization):ti,ab,kw  #8 ("hospitalization"):ti,ab,kw  #9 (time reduction):ti,ab,kw  #10 (time saving):ti,ab,kw  #11 (chair time):ti,ab,kw  #12 (HCP time):ti,ab,kw  #13 (#1 OR #2) AND (#3 OR #4) AND (#5 OR #6 OR #7 OR #8 OR #9 OR #10 OR #11 OR #12) |
| PubMed (MEDLINE) | ((((((((((((breast cancer[MeSH Terms]) OR (breast neoplasm[MeSH Terms])) OR (breast neoplasms[MeSH Terms])) OR (HER2-positive[Title/Abstract] AND breast cancer[Title/Abstract])) AND (((((((((drug administration routes[MeSH Terms]) OR (route*[Title/Abstract])) OR (frequenc*[Title/Abstract])) OR (administration[Title/Abstract])) OR (intraveno*[Title/Abstract])) OR (subcutan*[Title/Abstract])) OR (administration, intravenous[MeSH Terms])) OR (Injections, Subcutaneous[MeSH Terms])) OR (Infusions, Subcutaneous[MeSH Terms]))))) AND (((((((((((((((Health Care Rationing[MeSH Terms]) OR (healthcare utilization[Title/Abstract])) OR (healthcare resources utilization[Title/Abstract])) OR (healthcare resource utilization[Title/Abstract])) OR (hospitalization[Title/Abstract])) OR (length of stay[Title/Abstract])) OR (burden of disease[Title/Abstract])) OR (health care utilization[Title/Abstract])) OR (time reduction)) OR (resource use[Title/Abstract])) OR (time saving*[Title/Abstract])) OR (time-and-motion)) OR (time-saving*[Title/Abstract])) OR (HCP time)) OR (chair time))) AND (((((trastuzumab[Title/Abstract]) OR (pertuzumab[Title/Abstract])) OR ((trastuzumab[MeSH Terms]) OR (pertuzumab[Supplementary Concept]))))) |
| Virtual Health Library | ((pertuzumab) OR (trastuzumab)) AND (breast cancer) AND (healthcare resource utilization)  ((pertuzumab) OR (trastuzumab)) AND (breast cancer) AND (time saving)  ((pertuzumab) OR (trastuzumab)) AND (breast cancer) AND (time-and-motion) |

**Studies inclusion criteria**

*Preference outcomes*

▪ Observational studies with HER2-positive breast cancer patients and/or healthcare staff that assessed the preference for each route of drug administration;

▪ Clinical trials that included preference data from patients with HER2-positive breast cancer and/or healthcare staff who evaluated and compared preference according to each route of drug administration;

▪ Qualitative studies with patients with HER2-positive breast cancer and/or the health team that evaluated and compared the preference according to each route of drug administration through individual interviews or focus groups;

▪ Systematic literature reviews that directly or indirectly compared the preference of patients with HER2-positive breast cancer and/or the healthcare team according to each route of drug administration.

*Healthcare Resource Utilization Outcomes*

▪ Observational studies that assess which and how many health resources are used by patients with HER2-positive breast cancer for treatment in each route of drug administration;

▪ RSL that directly or indirectly compares which and how much health resources are used by patients with HER2-positive breast cancer in each route of drug administration.

**Excluded studies based on the full-text evaluation**

**Table S5.** List of excluded studies with reasons

| **Authors** | **Year** | **Title** | **Reason for exclusion** |
| --- | --- | --- | --- |
| Bartsch et al. | 2021 | Results from the FeDeriCa trial: are we reducing the burden of breast cancer treatment? | Comment publication |
| Belleudi et al. | 2019 | [Appropriate use of trastuzumab in Lazio Region: therapeutic scenarios and estimation of possible savings for the Regional Health Service.] | Did not present the outcome of interest |
| Cicin et al. | 2022 | An Open-Label, Multinational, Multicenter, Phase IIIb Study with Subcutaneous Administration of Trastuzumab in Patients with HER2-Positive Early Breast Cancer to Evaluate Patient Satisfaction | Did not present the comparison of interest |
| Ciruelos et al. | 2017 | Phase III trial to evaluate patient's preference for subcutaneous versus intravenous trastuzumab administration in patients with HER2 positive advanced breast cancer (ABC) under IV trastuzumab (IV-t) treatment for at least 4 months. ChangHER-SC study (GEICAM/2012-07) | Conference abstract |
| De Cock et al. | 2013 | Manual injection of subcutaneous trastuzumab vs intravenous infusion for HER2-positive early breast cancer: a time-and-motion study | Conference abstract |
| Dent et al. | 2019 | A multidisciplinary perspective on the subcutaneous administration of trastuzumab in HER2-positive breast cancer | Not a primary study - we collected studies from the reference list |
| Denys et al. | 2020 | Safety and tolerability of subcutaneous trastuzumab at home administration, results of the phase IIIb open-label BELIS study in HER2-positive early breast cancer | Did not present the outcome of interest |
| Duco et al. | 2020 | Trastuzumab/Hyaluronidase-oysk: A New Option for Patients With HER2-Positive Breast Cancer | Not a primary study - we collected studies from the reference list |
| DuMond et al. | 2021 | Fixed-dose combination of pertuzumab and trastuzumab for subcutaneous injection in patients with HER2-positive breast cancer: A multidisciplinary approach | Not a primary study - we collected studies from the reference list |
| Fallowfield et al. | 2015 | Implications of subcutaneous or intravenous delivery of trastuzumab; further insight from patient interviews in the PrefHer study | Did not present the comparison of interest |
| Fallowfield et al. | 2013 | Reasons for patients' preferences for subcutaneous or intravenous trastuzumab in the PrefHer study | Conference abstract |
| Franken et al. | 2020 | Hospital-based or home-based administration of oncology drugs? A micro-costing study comparing healthcare and societal costs of hospital-based and home-based subcutaneous administration of trastuzumab | Did not present the comparison of interest |
| Gregori et al. | 2022 | Time and motion study of a subcutaneous fixed-dose combination of pertuzumab and trastuzumab for the treatment of patients with HER2-positive early breast cancer (PHaTiMa) | Conference abstract |
| Inotai et al. | 2019 | Behind the subcutaneous trastuzumab hype: evaluation of benefits and their transferability to Central Eastern European countries | Not a primary study - we collected studies from the reference list |
| Jackisch et al. | 2015 | Subcutaneous Trastuzumab for HER2-positive Breast Cancer - Evidence and Practical Experience in 7 German Centers | Did not present the outcome of interest |
| Jackisch et al. | 2022 | White Paper on the Value of Time Savings for Patients and Healthcare Providers of Breast Cancer Therapy: The Fixed-Dose Combination of Pertuzumab and Trastuzumab for Subcutaneous Injection as an Example | Not a primary study - we collected studies from the reference list |
| Jin et al. | 2015 | The optimal choice of medication administration route regarding intravenous, intramuscular, and subcutaneous injection | Not a primary study - we collected studies from the reference list |
| Launay-Vacher, V. | 2013 | An appraisal of subcutaneous trastuzumab: a new formulation meeting clinical needs | Not a primary study - we collected studies from the reference list |
| Manevy F et al. | 2021 | Potential non-drug cost differences associated with the use of the fixed-dose combination of pertuzumab and trastuzumab for subcutaneous injection (PH FDC SC) in the treatment of HER2-positive early breast cancer patients in Western Europe and the United States. | Conference abstract |
| McCloskey et al. | 2022 | A Systematic Review of Time and Resource Use Costs of Subcutaneous Versus Intravenous Administration of Oncology Biologics in a Hospital Setting | Not a primary study - we collected studies from the reference list |
| O'Shaughnessy et al. | 2020 | Patient (pt) preference for the pertuzumab-trastuzumab fixed-dose combination for subcutaneous use (PH FDC SC) in HER2-positive early breast cancer (EBC): primary analysis of the open-label, randomised crossover PHranceSCa study | Conference abstract |
| O'Shaughnessy et al. | 2020 | 80O Patient (pt) preference and satisfaction with the subcutaneous fixed-dose combination of pertuzumab (P) and trastuzumab (H) in pts with HER2-positive early breast cancer (HER2+ eBC): interim analysis of the open-label, randomised cross-over PHranceSCa study | Conference abstract |
| Pailler, C. | 2019 | [Experience of patients treated at home with trastuzumab] | Did not present the outcome of interest |
| Papadmitriou et al. | 2015 | The socio-economical impact of intravenous (IV) versus subcutaneous (SC) administration of trastuzumab: future prospectives | Not a primary study - we collected studies from the reference list |
| Pivot et al. | 2016 | Patient preference of trastuzumab administration (SC versus IV) in HER2-positive metastatic breast cancer: results of the randomised Metaspher study | Conference abstract |
| Pivot et al. | 2016 | Efficacy and safety of subcutaneous trastuzumab and intravenous trastuzumab as part of adjuvant therapy for HER2-positive early breast cancer: final analysis of the randomised, two-cohort PrefHer study | Conference abstract |
| Pivot et al. | 2013 | Patient preference for subcutaneous versus intravenous trastuzumab: results of the prefher study | Same data as Pivot et al., 2014 |
| Pivot et al. | 2013 | Patient preference for subcutaneous trastuzumab via handheld syringe versus intravenous infusion in HER2-positive early breast cancer: cohort 2 of the PrefHer study | Same data as Pivot et al., 2014 |
| Pivot et al. | 2017 | Efficacy and safety of subcutaneous trastuzumab and intravenous trastuzumab as part of adjuvant therapy for HER2-positive early breast cancer: Final analysis of the randomised, two-cohort PrefHer study | Did not present the outcome of interest |
| Pivot et al. | 2014 | Patients' preferences for subcutaneous trastuzumab versus conventional intravenous infusion for the treatment of HER2-positive early breast cancer: final analysis of 488 patients in the international, randomised, two-cohort PrefHer study | Conference abstract |
| Pivot et al. | 2013 | Preference for subcutaneous or intravenous administration of trastuzumab in patients with HER2-positive early breast cancer (PrefHer): an open-label randomised study | Same data as Pivot et al., 2014 |
| Ponzetti et al. | 2016 | Potential resource and cost saving analysis of subcutaneous versus intravenous administration for rituximab in non-Hodgkin's lymphoma and for trastuzumab in breast cancer in 17 Italian hospitals based on a systematic survey | Did not present the outcome of interest |
| Reinisch et al. | 2020 | 86P Patients (pts) preference for different administration methods of trastuzumab (T) in pts with HER2+ early breast cancer (BC) treated within the GAIN-2 trial | Conference abstract |
| Roe, H. | 2014 | Patient's perception of a nurse-led Trastuzumab pathway | Did not present the outcome of interest |
| Tan et al. | 2021 | Fixed-dose combination of pertuzumab and trastuzumab for subcutaneous injection plus chemotherapy in HER2-positive early breast cancer (FeDeriCa): a randomised, open-label, multicentre, non-inferiority, phase 3 study | Did not present the outcome of interest |
| Tjalma et al. | 2017 | The smooth and bumpy road of trastuzumab administration: from intravenous (IV) in a hospital to subcutaneous (SC) at home | Not a primary study - we collected studies from the reference list |
| Triantafyllidi, E. and Triantafillidis, JK. | 2022 | Systematic Review on the Use of Biosimilars of Trastuzumab in HER2+ Breast Cancer | Did not present the outcome of interest |
| Valachis et al. | 2019 | Use of subcutaneous and intravenous trastuzumab: real-world experience from three hospitals in Sweden | Did not present the outcome of interest |
| Waller et al. | 2021 | Intravenous and subcutaneous formulations of trastuzumab, and trastuzumab biosimilars: implications for clinical practice | Not a primary study - we collected studies from the reference list |
| Swain et al. | 2023 | Incidence and severity of anaphylaxis and hypersensitivity in trials of intravenous pertuzumab plus trastuzumab or the fixed-dose combination of pertuzumab and trastuzumab for subcutaneous injection for HER2-positive breast cancer | Did not present the outcome of interest |
| Franken et al. | 2018 | Potential cost savings owing to the route of administration of oncology drugs: a microcosting study of intravenous and subcutaneous administration of trastuzumab and rituximab in the Netherlands. | Did not present the outcome of interest |
| De La Vega et al. | 2017 | Economic impact of the introduction of subcutaneous trastuzumab in the pharmacotherapeutic guide. | Did not present the outcome of interest |
| Mylonas et al. | 2017 | Cost minimization analysis of Herceptin subcutaneous versus herceptin intravenous treatment for patients with HER2+ breast cancer in Greece. | Conference abstract |
| Alcântara et al. | 2021 | Jornada da paciente e levantamento dos custos do acompanhamento do câncer de mama inicial e metastático no Sistema Único de Saúde (SUS) | Did not present the comparison of interest |
| Gligorov et al. | 2017 | Switching between intravenous and subcutaneous trastuzumab: Safety results from the PrefHer trial. | Did not present the outcome of interest |
| Manevy et al. | 2021 | Potential non-drug cost differences associated with the use of the fixed-dose combination of pertuzumab and trastuzumab for subcutaneous injection (PH FDC SC) in the treatment of HER2-positive early breast cancer patients in Western Europe and the United States. | Conference abstract |

**Risk of Bias Assessment**

**Figure S1.** Risk of bias of observational studies by the Risk Of Bias In Non-randomised Studies - of Interventions (ROBINS-I).


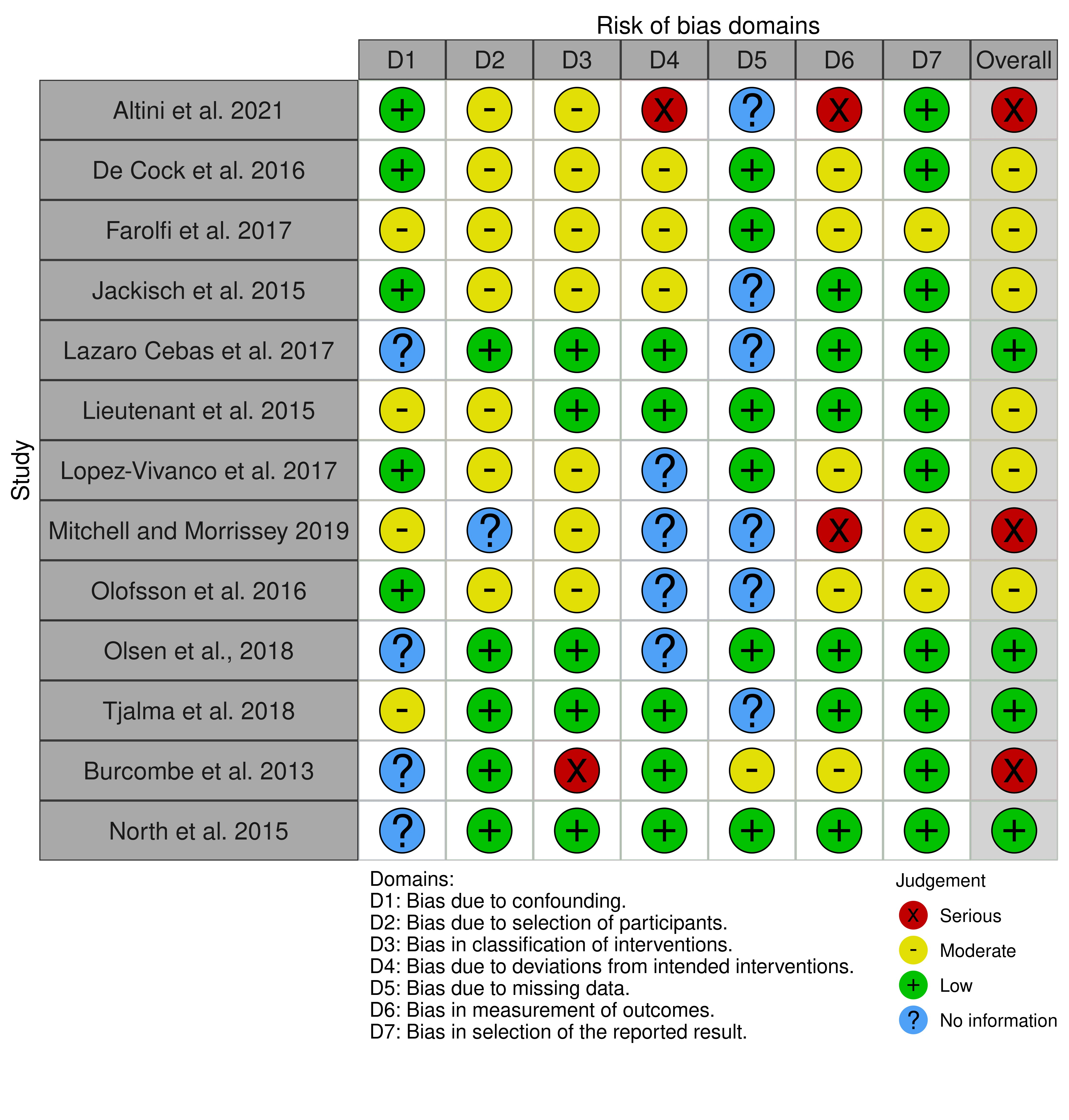


**Figure S2.** Risk of bias of randomized controlled trials by the Risk Of Bias 2 (RoB 2).


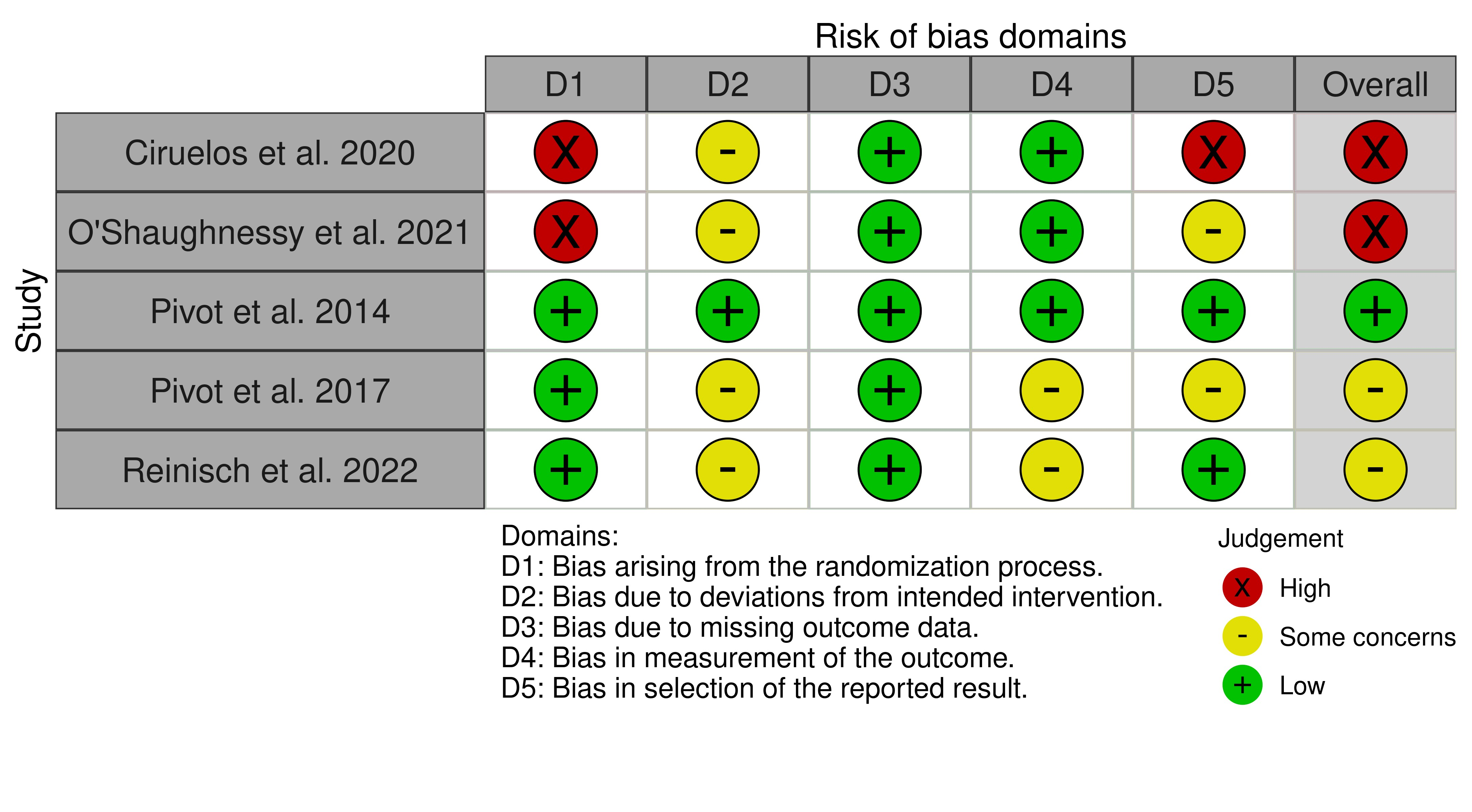


**Table S6.** Methodology assessment of economic studies by the Consolidated Health Economic Evaluation Reporting Standards 2022 (CHEERS 2022).

| **Section/topic** | **Item No** | **Guidance for reporting** | **Castro-cordero, 2019** | **Elsamany, 2020** | **Hedayati, 2019** | **Kashiura, 2019** | **O'Brien, 2019** | **Rojas, 2020** |
| --- | --- | --- | --- | --- | --- | --- | --- | --- |
| **Title** | | | | | | | | |
| Title | 1 | Identify the study as an economic evaluation and specify the interventions being compared. | Title, Page 1 | Title, Page 1 | Title, Page 1 | Title, Page 1 | Title, Page 1 | Title, Page 1 |
| **Abstract** | | | | | | | | |
| Abstract | 2 | Provide a structured summary that highlights context, key methods, results, and alternative analyses. | Page 1 | Page 2 | Page 1 | Page 1 | Page 1 | Page 1 |
| **Introduction** | | | | | | | | |
| Background and objectives | 3 | Give the context for the study, the study question, and its practical relevance for decision making in policy or practice. | Page 2 | Page 3 | Page 2, First Paragraph | Introduction, First Paragraph | Introduction, First Paragraph | Introduction, First Paragraph |
| **Methods** | | | | | | | | |
| Health economic analysis plan | 4 | Indicate whether a health economic analysis plan was developed and where available. | Methods, Line 10 | Methods, First Paragraph | Methods, First Paragraph | Methods, First Paragraph | Methods, Third Paragraph | Methods, Second Paragraph |
| Study population | 5 | Describe characteristics of the study population (such as age range, demographics, socioeconomic, or clinical characteristics). | Not Applicabe | Not Applicabe | Methods, Third Paragraph | Methods, Second Paragraph | Not Applicabe | Not Applicabe |
| Setting and location | 6 | Provide relevant contextual information that may influence findings | Resumen, Line 7 | Introduction, First Paragraph | Introduction, First Paragraph | Methods, First Paragraph | Introduction, First Paragraph | Methods, Second Paragraph |
| Comparators | 7 | Describe the interventions or strategies being compared and why chosen. | Methods, Line 7 | Methods, First Paragraph | Methods, First Paragraph | Methods, Tenth Paragraph | Methods, First and Second Paragraph | Methods, Line 6 |
| Perspective | 8 | State the perspective(s) adopted by the study and why chosen. | Resumen, Line 7 | Abstract, First Paragraph | Results, Ninth Paragraph | Page 4, Sixth Paragraph | Introduction, Last Paragraph | Introduction, Line 23 |
| Time horizon | 9 | State the time horizon for the study and why appropriate. | Results, Fifth Paragraph | Background, Last Paragraph | Methods, Second Paragraph | Page 4, Twelfth Paragraph | Methods, Fourth Paragraph | Methods, Third Paragraph |
| Discount rate | 10 | Report the discount rate(s) and reason chosen. | Methods, Second Paragraph | Not Reported | Not Reported | Not Reported | Results, Third Paragraph | Methods, Third Paragraph |
| Selection of outcomes | 11 | Describe what outcomes were used as the measure(s) of benefit(s) and harm(s). | Methods, Line 1 | Results, First Paragraph | Methods, Sixth Paragraph | Results, First Paragraph | Results, First and Second Paragraph | Results, First Paragraph |
| Measurement of outcomes | 12 | Describe how outcomes used to capture benefit(s) and harm(s) were measured. | Methods, Second Paragraph | Discussion, First Paragraph | Methods, Sixth Paragraph | Methods, Figure 2 | Results, First and Second Paragraph | Discussion, First Paragraph |
| Valuation of outcomes | 13 | Describe the population and methods used to measure and value outcomes. | Not Applicabe | Not Applicabe | Methods, Third Paragraph | Results, First Paragraph | Not Applicabe | Not Applicabe |
| Measurement and valuation of resources and costs | 14 | Describe how costs were valued. | Methods, Table 1 and 2 | Page 25 | Methods, Table 1 | Discussion, Table 4 | Page 4, Table 2 | Methods, Table 1 |
| Currency, price date and conversion | 15 | Report the dates of the estimated resource quantities and unit costs, plus the currency and year of conversion. | Not Reported | Not Reported | Not Reported | Not Reported | Table 2, Appendix | Not Reported |
| Rationale and description of model | 16 | If modeling is used, describe in detail and why used. Report if the model is publicly available and where it can be accessed. | Not Applicabe | Discussion, First Paragraph | Not Applicabe | Methods, First Paragraph | Methods, Third Paragraph | Methods, Second Paragraph |
| Analytics and assumption | 17 | Describe any methods for analyzing or statistically transforming data, any extrapolation methods, and approaches for validating any model used. | Methods, Bullet Points | Results, Last Paragraph | Discussion, First Paragraph | Methods, Last Paragraph | Page 5, Line 10 | Methods, Last Paragraph |
| Characterising heterogeneity | 18 | Describe any methods used for estimating how the results of the study vary for subgroups. | Not Reported | Not Reported | Not Reported | Not Reported | Not Reported | Not Reported |
| Characterising distributional effects | 19 | Describe how impacts are distributed across different individuals or adjustments made to reflect priority populations. | Not Applicabe | Abstract, Line 9 | Methods, Ninth Paragraph | Discussion, Table 4 | Discussion, Last Paragraph | Not Applicabe |
| Characterising uncertainty | 20 | Describe methods to characterize any sources of uncertainty in the analysis. | Not Reported | Methods, Last Paragraph | Not Reported | Not Reported | Discussion, Fifth Paragraph | Not Reported |
| Approach to engagement with patients and others affected by the study | 21 | Describe any approaches to engage patients or service recipients, the general public, communities, or stakeholders (such as clinicians or payers) in the design of the study. | Not Applicabe | Not Applicabe | Not Applicabe | Introduction, Last Paragraph | Not Applicabe | Not Applicabe |
| **Results** | | | | | | | | |
| Study parameters | 22 | Report all analytic inputs (such as values, ranges, references) including uncertainty or distributional assumptions. | Not Reported | Not Reported | Not Reported | Not Reported | Not Reported | Methods, Last Paragraph |
| Summary of main results | 23 | Report the mean values for the main categories of costs and outcomes of interest and summarize them in the most appropriate overall measure. | Table 2 and 3 but information is missing | Page 25 | Methods, Table 1 | Discussion, Table 4 | Table 2 | Discussion, Table 5 and 6 |
| Effect of uncertainty | 24 | Describe how uncertainty about analytic judgments, inputs, or projections affect findings. Report the effect of choice of discount rate and time horizon, if applicable | Not Reported | Not Reported | Not Reported | Results, Last Paragraph | Discussion, Fifth Paragraph | Not Reported |
| Effect of engagement with patients and others affected by the study | 25 | Report on any difference patient/service recipient, general public, community, or stakeholder involvement made to the approach or findings of the study | Not Applicabe | Not Applicabe | Not Applicabe | Page 4 | Not Applicabe | Not Applicabe |
| **Discussion** | | | | | | | | |
| Study findings, limitations, generalisability, and current knowledge | 26 | Report key findings, limitations, ethical or equity considerations not captured, and how these could affect patients, policy, or practice. | Discussion, Fourth Paragraph Table 1, 2 and 3 | Discussion, Sixth Paragraph | Methods, Table 1 Results, Figure 1 | Discussion, Last Paragraph | Discussion, Fifth Paragraph | Discussion, Fifth Paragraph |
| **Other relevant information** | | | | | | | | |
| Source of funding | 27 | Describe how the study was funded and any role of the funder in the identification, design, conduct, and reporting of the analysis | Page 1 | Page 13 | Page 1 | Page 1 | Page 9 | Page 1 |
| Conflicts of interest | 28 | Report authors conflicts of interest according to journal or International Committee of Medical Journal Editors requirements. | Not Reported | Page 15 | Page 2 | Page 1 | Page 9 | Page 1 |

**Table S7.** Characteristics of the included studies related to healthcare resource utilization.

| **authors, year** | **country** | **study design** | **diagnose** | **therapy** | **regimen** | **measures** | **sample size** | **Resource-savings** | **Cost-savings** | |
| --- | --- | --- | --- | --- | --- | --- | --- | --- | --- | --- |
| Simoens et al., 2021 (Simoens et al., 2021) | Belgium | Case study - Belgium healthcare center | HER2-overexpressing breast cancer | IV biosimilar trastuzumab SC reference trastuzumab (Herceptin®, Roche) | 18 cycles of adjuvant trastuzumab (1 year) | -Drug costs for a 1 year course of adjuvant treatment; -Healthcare costs (i.e., drug costs, HCP time costs and consumables costs) | 100 | NR | **Drug costs:** -IV treatment was less expensive than with SC for patients weighing up to 75 kg. **Healthcare costs:** -IV treatment was less expensive than with SC for a patient weighing up to 62.5 kg; -IV costs exceeded those with SC for a patient weighing more than 75 kg because IV treatment is dosed on a mg/kg basis and the SC formulation has a fixed dose for all body weights. **Drug and healthcare costs:** -Estimates costs to a sample of 100 patients: lower drug costs with IV treatment compared to SC offset higher healthcare provider time and consumable costs. | |
| De Cock et al., 2016 (De Cock et al., 2016) | Canada, France, Switzerland, Denmark, Italy, Russia, Spain, Turkey | Multinational, multicenter, observational T&M study [PrefHer (NCT01401166)] | HER2-positive early breast cancer | Trastuzumab | -Neoadjuvant chemotherapy followed by SC trastuzumab (600 mg) for 18 cycles followed by IV (standard dosing) compared with the reverse; -Cohort 1: SC by injection device; -Cohort 2: SC by handheld syringe. | -Patient chair time per session; -Active HCP time per session | 8 healthcare centers | **Time-saving for patient chair per session:** -SC SID saved a mean of 57 min (range across countries: 47–86; P < 0.0001) versus IV; -SC HHS saved a mean of 55 min (range across countries: 40–81; P < 0.0001) versus IV.  **Time-saving for active HCP time per session:**  -SC SID reduced by a mean of 13 min (range across countries: 4–16; P < 0.0001) versus IV; -SC HHS reduced by a mean of 17 min (range across countries: 5–28; P < 0.0001) versus IV. | NR | |
| Castro-Cordero et al., 2019 (Cordero et al., 2019) | Costa Rica | Cost-minimization study | HER2-overexpressing breast cancer | Trastuzumab | -SC: 17 consecutive doses with a fixed dose of 600 mg; -IV: 4 mg/kg and 16 subsequent doses calculated at 2 mg/kg. | Direct costs:  -Costs for each route (IV: USD 1,800.00; SC: USD 1,602.00); -Cost per mg (USD 4.1; SC: 2.7); -Treatment costs per patient (IV: USD 29,782.00; SC: 27,234.00) | NR | **Patient's perspective - time for application:** -SC: 20 minutes; IV: 1 hour  -SC represents a 45% reduction in administration time per session, per patient, compared to IV. **The annual estimate of expected cases using SC:** -1st year: expand care coverage to 46 additional patients -5th year: expand care coverage to 56 additional patients | **The economic evaluation - cost per application:** -SC: USD 78.6; IV: USD 467.3; **Financial point of view:** -SC option represents the lowest cost (USD 4,000.0 per treatment, per patient); **Future savings:**  -For the next five years: USD 7.7 million for the SC application compared to IV | |
| Mitchell and Morrissey, 2019 (Mitchell and Morrissey, 2019) | United Kingdom | Cross-sectional | HER2-positive early breast cancer | Trastuzumab | NR | -Patient chair time per session; -Direct costs for each administration route | 116 | **IV:** Longer duration of administration led to a more frequent need for alternative methods of intravenous access [includes the cost of consumables such as PICCs, CVCs, and ports, ad hoc visits to the hospital procedure team, and increased hospital admissions due to infections].  **Patient chair time per session:** -IV: 2-hour -SC: 30-minute | **Total cost:** -IV: USD 3,949.53 -SC: USD 1,359.57 | |
| Olofsson et al., 2016 (Olofsson et al., 2016) | Sweden | Cross-sectional | HER2-positive early or metastatic breast cancer | Trastuzumab | -SC: 600 mg fixed dose; -IV: 578 mg per first-time treatment and 434 mg per subsequent treatment. | -Direct costs -Questionnaires: **Patients:** background questions (e.g. age, occupation), questions about the disease and treatment, transportation, and opinion about trastuzumab treatment; **Nurses:** questions about the patient and the time and resources allocated to trastuzumab treatment | 101 | **Time allocated to treatment:** -The average nurse time was shorter for SC compared to IV: the time difference was 17 min for first-time visits (p = 0.0026), and 14 min for subsequent treatment occasions (p < 0.0001); -Nurses spent less time on initiation and termination with SC patients; -IV allocated more time to treatment compared to SC: difference in time spent at the hospital, when compared IV to SC, was 101 min for first-time visits (p = 0.0499), and 23 min for subsequent treatments (p = 0.0033). **Work absenteeism and transportation:** -IV had taken more time off from work than SC (IV: 14%; SC: 5%, p = 0.0223) -Time off from work for subsequent patients: IV - 33 min; SC - 12 min -Time for the accompanying kin: IV - 22 min; SC - 21 min | **Societal treatment costs:** -First-time treatment occasion: IV - USD 3,201.39; SC - USD 2,237.96 = cost decrease of USD 963.43 for SC per visit; -Subsequent treatment occasion: IV - USD 2,259.49; SC - USD 2,134.62 = a cost decrease of USD 125.95 for SC per visit; -Drug waste associated with the preparation of IV: cost reduction of USD 105.49 per first-time visit and USD 78.58 per subsequent visit. | |
| Lieutenant et al., 2015 (Lieutenant et al., 2015) | France | Cross-sectional | HER2-overexpressing breast cancer | Trastuzumab | -SC: NR; -IV: first infusion of 8 mg/kg over 90 min, then the maintenance of 6 mg/kg in 30 minutes, every 3 weeks. | -Time savings; -Direct costs | 8 | **Prescriptions of the administration:** -IV: 138 min for the loading dose and 76 min for the maintenance dose -SC: 32.5 min = reduction of 77% and 58% of the time compared to the IV **Time savings:** *Transit time:* -IV: 90 to 30 min -SC: 5 min *Manufacturing time:* -IV: 10.25 to 9.5 min -SC: 3 min *Annual staff time:* SC saves 1,956.22 hours compared to IV [the main professional categories impacted by this time saving are hospital assistants and nurses] *Time spent in the hospital for the patient (“chair occupation time”):* Potential time savings versus loading dose (in minutes): SC saves 105.5 minutes compared to IV Potential time savings compared to the maintenance dose (in minutes): SC saves 43.5 minutes compared to IV | **Financial gain linked to the SC route:** USD 23,778.56/year | |
| O'Brien et al., 2019 (O’Brien et al., 2019) | Ireland | Cross-sectional and micro-costing study | HER2-overexpressing breast cancer | Trastuzumab | -SC: 600 mg fixed dose; -IV: 150 mg (average patient weight of 72.05 kg)  17 triweekly dosing cycles (1 year of treatment/a full treatment cycle) | Direct and indirect costs | -Hospital 1 (Nurse-led Clinic): 431-inpatient and 85-day procedure beds -Hospital 2 (Infusion Clinic): 192 beds and caters for up to 38,400 admissions and 72,500 outpatient attendances each year | **Patient treatment room time:** -IV = 3052 seconds (50.87 min); SC = 841 seconds (14 min). | **Costs of Consumables:** -Per treatment cycle: IV = USD 60.58; SC = USD 27.89; saving of USD 32.69 with SC excluding the drug costs; -For a complete 17-cycle treatment: IV = USD 1,029.91; SC = USD 474.15; saving of USD 555.77 per patient with SC. **HCP Costs (Preparation and administration):** -Per treatment cycle: IV = USD 48.37; SC = USD 10.58; -For a complete 17-cycle treatment: IV = USD 822.21; SC = USD 179.89; saving of USD 642.32 with SC. **HCP Costs (extrapolating these results to a hospital treating 25 patients per year and hospitals):** -IV = USD 20,555.27; SC = USD 4,497.18; an average saving of USD 16,058.09 (78%) in favor to SC. **Drug Costs:** -Total cost of a 17-cycle treatment: IV = USD 37,567.35; SC = USD 37,032.34; saving of USD 535.00 with SC. **Indirect Costs:** -Lost productivity (17-cycle treatment per patient): IV = USD 262.38 (loss of 2.15 working days); SC = USD 72.28 (loss of 0.60 working days). **Total Cost (full 17-cycle treatment):** **-Direct costs: savings of USD 1,733.09 in favor of SC; -Direct+Indirect: savings of USD 1,923.18 in favor of SC.** | |
| Lopez-Vivanco et al., 2017 (Lopez-Vivanco et al., 2017) | Spain | Cross-sectional, T&M and micro-costing [PrefHer (NCT01401166)] | HER2-positive early breast cancer | Trastuzumab | -Neoadjuvant chemotherapy followed by SC trastuzumab (600 mg) for 18 cycles followed by IV (standard dosing) compared with the reverse; -Cohort 1: SC by injection device; -Cohort 2: SC by handheld syringe. | T&M: -HCP time (preparation and administration); -Patient infusion chair time; -Patient treatment room time; -Patient hospital time. Costs: -Direct costs (costs of HCP, consumables, and drugs); -Indirect costs (lost productivity). | 307 (IV: 159; SC: 148) | **HCP time:** -SC: 13.2 min (95% CI 8.9–17.5); IV: 27.2 min (95% CI 21.8–32.6); mean relative reduction: >50%; -SC: absolute time-savings = 3.6 to 22.7 min; relative time-savings = 17 and 66%; -Time-saving on drug preparation and administration processes, nursing time (IV: 21.8 vs. SC: 11.2 min), pharmacist time (IV: 4.2 vs. SC: 1.2 min), and nursing assistant time (IV: 1.1 vs. SC: 0.8 min); -In the treatment room: IV: 8.2 h (95% CI 6.5–9.8); SC: 4 h (95% CI 2.7–5.2); to treat ten patients per year, replacing IV by SC: annual savings of 42 h. **Patient chair time, treatment room time, and hospital time:** -Patient chair time: IV: 101 min; SC: 20 min; reduction of 80%; -Patient treatment room time: IV: 120 min; SC: 30 min; reduction of 45%; -Patient hospital time: IV: 205 min; SC: 115 min.; reduction of 44%. | **Direct costs** *Costs of tasks:* -Preparation and administration/cycle: IV: USD 13.74; SC: USD 6.47; -Preparation and administration/complete 18-cycle treatment: IV: USD 247.26; SC: USD 116.40; savings of USD 130.87 in favor of SC. *Costs of consumables:* -Per treatment cycle: IV: USD 9.32; SC: USD 2.58; savings of USD 6.74 in favor of SC; -Complete 18-cycle treatment: IV: USD 167.63; SC: USD 46.31; saving of USD 121.33 per patient in favor of SC. *Drug costs (18-cycle treatment):* -IV: USD 31,320.60; SC: USD 30,516.73; savings of USD 803.88 in favor of SC. **Indirect costs (lost productivity):** -By patient room time: IV: USD 219.73 (loss of 4.5 working days); SC: USD 54.93 (loss of 1.1 working days); -By hospital time: IV: USD 375.37 (loss of 7.7 working days); SC: USD 210.58 (loss of 4.3 working days);  - SC resulted in a reduction in indirect costs of USD 164.80 per patient compared with IV. **Total cost (full 18-cycle treatment):** -Direct costs: savings of USD 1,056.29 in favor of SC; **-Direct+Indirect:** savings of USD 1,221.09 in favor of SC. | |
| North et al., 2015 (North et al., 2015) | New Zeland | Noninterventional, descriptive and cost-minimization study [SafeHer (NCT01566721)] | HER2-positive early breast cancer | Trastuzumab | -SC: 600 mg fixed dose -IV: NR 17 triweekly dosing cycles (1 year of treatment/a full treatment cycle) | T&M: -HCP time (preparation and administration). Costs: -Direct costs (costs of HCP, consumables, and drugs). | 18 (IV: 12; SC: 6) | **HCP time:** -IV: 13.02 min; SC: 6.90 min; time-savings of 43%.  **Chair time:** -IV: 47.44 min; SC: 10.49 min; time-savings of 75%.  **Overall time to prepare and deliver trastuzumab therapy:** -Markedly lower for the SC vs IV formulation: average HCP nurse time, chair cost, and pharmacist time; | **HCP time:** -Cost savings of USD 2.85 in favor of SC.  **Chair time:** -Cost savings of USD 24.86 in favor of SC.  **Overall time to prepare and deliver trastuzumab therapy:** -Time savings of USD 38.20 in favor of SC; consumables cost savings: USD 9.48/administration; -Estimated cost saving by switching to the SC: USD 47.78/patient/cycle. | |
| Burcombe et al., 2013 (Burcombe et al., 2013) | United Kingdom | Non-interventional, prospective, multi-centre descriptive study [PrefHer (NCT01401166)] | HER2-positive early breast cancer, adjuvant | Trastuzumab | -Neoadjuvant chemotherapy followed by SC trastuzumab (600 mg) for 18 cycles followed by IV (standard dosing) compared with the reverse; -Cohort 1: SC by injection device; -Cohort 2: SC by handheld syringe. | T&M: -HCP time (preparation and administration); -Patient chair time. Costs: -Direct costs (costs of HCP, consumables, and drugs). | 24 (12 in each group) | **Time-savings:** -HCP time administration and preparation: SC: 24.6 min; IV: 92.6 min; -Patient time in the unit of care: SC: 30.3 min; IV: 94.5 min. The course of 18 infusions: additional patient time of 19 hours and 16 minutes in favor of IV.  -Patient chair time for administration: SC: 19.8 min; IV: 75.0 min. | **Costs/patient episode (administration and preparation):** -SC: USD 35.74; IV: USD 156.30; cost-savings: USD 120.55 in favor of SC. | |
| Tjalma et al., 2018 (Tjalma et al., 2018) | Belgium | Observational, non-interventional, prospective, monocentric time, motion, and cost assessment study | HER2-positive early or metastatic breast cancer | Trastuzumab | -SC: 600 mg fixed dose -IV: initial loading dose is given at 8 mg/kg of body weight over 90 min IV infusion, followed by 6 mg/kg over 30–90 min IV infusion every three weeks for 52 weeks; -Adjuvant or neoadjuvant trastuzumab up to 1 year: 18 triweekly dosing cycles (1 year of treatment/a full treatment cycle) | T&M: -HCP time (preparation and administration); -Patient infusion chair time; -Patient hospital time. Costs: -Direct costs (costs of HCP, consumables, and drugs). | 130 | **Active HCP time:**  -SC: 13.9 min (95% CI, 12.6–15.1 min); IV: 67.6 min (95% CI, 64.6–70.7 min) + 9.8 additional minutes for completion of the preparation tasks that were performed at the hospital pharmacy; **Patient hospital time:**  -IV: 172.7 min; SC: 50.2 min; 18 treatment cycles of IV: additional patient time of 36,9 h; **Patient chair time:**  -IV: 136.7 min; SC: 10.6 min. | **Cost saving:** -Overall costs: SC: USD 11.43; IV: USD 242.04; -HCP time per patient episode: IV: USD 40.33; SC: USD 8.52; -Cost of consumables (syringes, cotton, alcohol, etc.): IV: USD 25.45/patient episode; SC: USD 2.91/administration; -Drug wastage: IV administration represents a significant additional cost: USD 149.87 (N = 10; invoices), USD 175.24 (N = 65; for the three-weekly regimen); SC: cost saving of USD 229.58/administration or USD 4,132.50/treatment cycle. | |
| Altini et al., 2020 (Altini et al., 2020) | Italy | Pilot, observational and retrospective study | HER2-overexpressing breast cancer | Trastuzumab | -SC: 600 mg fixed dose; -IV: variable doses depending on the weight or body surface of the patient and by long administration times. | T&M: -Patient infusion chair time; -Patient treatment room time. Costs: -Direct costs; -Speculative costs (scenarios). | 314 (2,229 accesses) | **Time to therapy completion by administration route:** -Average total time per patient: IV: 02h37min; SC: 01h19min; saving of 01h18min in favor of SC; -Time spent seated in treatment chair: IV: 01h09min; SC: 33min; saving of 36min in favor of SC. | **Costs (antineoplastic drug, healthcare personnel, catheter, consumables, possible waste of the drug, and structural costs):** -SC: USD 1,847.21; IV: USD 1,934.08; **Scenarios of costs:** -IV alone: USD 4,5 million; -Greater use of SC (from 22% to 96%): USD 147,689.61 | |
| Elsamany et al., 2020 (Elsamany et al., 2020) | Saudi Arabia | Budget impact model study | HER2-overexpressing breast cancer | Trastuzumab | -SC: 600 mg fixed dose; -IV: variable doses depending on the weight or body surface of the patient and by long administration times. | -Drug and non-drug costs (direct and indirect) in the two arms annually over a 3-year time horizon; -Two scenarios: 1st scenario (similar to that adopted in King Abdullah Medical city), evaluated the impact of gradual replacement of IV formulation by SC in up to 75% of patients (SC adoption in Year 1: 25%; Year 2: 50%; Year 3: 75%), 2nd scenario, the evaluated impact of replacing IV formulations by SC in 100% patients in year 1, 2 and 3; -HCP time. | 394 (1182 total patients over 3 years) | NR | **Costs to prepare and administer the drugs formulations over 3 years:** -SC: USD 83,586.20; IV: USD 1,003,033.59; savings of USD 919,447.39 (USD 777.91/patient/year) in favor of SC. **Total annual costs (drug and non-drug costs):** -1st scenario: IV: USD 98 million; SC: USD 79 million; total budget savings: USD 19,181,858 after 3 years in favor of SC (drug costs: USD 80 million; non-drug costs: USD 1.01 million); -2nd scenario: SC: USD 60 million; IV: USD 98 million; total budget savings: USD 36,363,717 after three years in favor of SC (drug costs: USD 16.3 million; non-drug costs: USD 2.05 million). **Indirect costs (lost productivity):** -SC: USD 26,863.37; IV: USD 682,646.94; total savings/productivity gain: USD 655,783.30 for total patients over three years in favor of SC. | |
| Cebas et al., 2016 (Cebas et al., 2016) | Spain | Cross-sectional questionnaire-based study | HER2-overexpressing breast cancer | Trastuzumab | SC: 600 mg fixed dose; IV: a loading dose of 8mg/kg and subsequent doses of 6 mg/kg; | Financial impact: -Number of preparations; -Drug cost; -Consumables used for preparation and administration; -Nursing staff time for preparation. | 76 | **Total time saved by the nursing staff for preparation:** 78.4h in favor of SC. | **Total cost:** -SC: USD 246.12; IV: USD 254.00. **Consumables used for preparation and administration:**  -Annual savings: USD 9.41 in favor of SC. | |
| Olsen et al., 2017 (Olsen et al., 2017) | Denmark | Cross-sectional | HER2-overexpressing breast cancer | Trastuzumab | SC: 600 mg fixed dose; IV: a loading dose of 8mg/kg and subsequent doses of 6 mg/kg; 17 triweekly dosing cycles (1 year of treatment/a full treatment cycle) | -Time consumption/cycle; -Costs with patient's time consumption. | 76 | **Time consumption/cycle:** time consumption for nurses and laboratory technicians and the patients’ time consumption is markedly higher for IV compared to SC (reasons: infusion time for IV is longer than the injection time for SC and the preparation of the infusion fluid at the hospital pharmacy takes a longer time than the preparation of the SC syringe). | **Costs:** Excluding patient’s time consumption: cost-saving of USD 258.50 in favor of SC; Including patient’s time consumption: cost-saving of USD 288.85 in favor of SC. | |
| Kashiura et al., 2019 (Kashiura et al., 2019) | Brazil | Budget impact model study [HANNAH (NCT00950300)] | HER2-positive early or metastatic breast cancer | Trastuzumab | -8 Cycles neoadjuvant chemotherapy with concurrent trastuzumab; -3-Weekly standard IV compared with SC trastuzumab (600 mg); -Adjuvant or neoadjuvant trastuzumab up to 1 year | Hypothetical incorporation of trastuzumab SC in the private setting. | 31,589 | NR | **Resource-saving over five years with the incorporation of trastuzumab SC:** USD 183,166,915.67 (USD 2,769,941.86, USD 290,672.56, and USD 48,674.50 for large, medium, and small healthcare plans, respectively); **Incorporation of SC:**  -HER-2 positive early breast cancer: savings of up to USD 176,859,259.46; -HER-2 positive metastatic breast cancer: savings of up to USD 6,307,656.20. | |
| Farolfi et al., 2017 (Farolfi et al., 2017) | Italy | Retrospective cohort study [PrefHer (NCT01401166)] | HER2-positive early breast cancer | Trastuzumab | -Neoadjuvant chemotherapy followed by SC trastuzumab (600 mg) for 18 cycles followed by IV (standard dosing) compared with the reverse; -Cohort 1: SC by injection device; -Cohort 2: SC by handheld syringe. | Direct and indirect costs | 114 | **Preparation time/patient:** -IV: 10.5 h; SC: 3.0 h (-71.7% compared to IV); SC<-->IV: 3.7 h (p<0.001 for the comparison of these three scenarios); **Administration time/patient:**  -IV: 6.4 h; SC: 0.7 h (-89.3% compared to IV); SC<-->IV: 3 h (-47% compared to IV)(p<0.001 for the comparison of these three scenarios); | **Direct costs:**  -Total cost of the drugs: IV: USD 1,665,559.84; SC: USD 1,739,771.47; SC<-->IV*: USD 1,738,435.52; -Costs/patient: IV: USD 14,723.50; SC: USD 15,261.55; SC<-->IV: USD 15,252.92 (p = 0.832 for the comparison of these three scenarios). **Indirect costs:** Outpatient clinic costs/patient: IV: USD 620.88; SC: USD 66.32; SC<-->IV: USD 328.63 (p<0.001 for the comparison of these three scenarios). **Direct + Indirect costs:** **-Costs/patient: IV: USD 15,346.95; SC: USD 15,389.43; SC<-->IV: USD 15,672.69 (p = 0.959 for the comparison of these three scenarios).** | |
| Hedayati et al., 2019 (Hedayati et al., 2019) | Sweden | Retrospective study (economic efficiency study) | HER2-overexpressing breast cancer | Trastuzumab | SC: 600 mg fixed dose; IV: a loading dose of 8mg/kg and subsequent doses of 6 mg/kg every 3-weeks; | -Time savings; -Direct costs | 178 | **Time savings for nurses:**  - Administration: 1st session - IV: 90min; SC: 10min; subsequent sessions - IV: 30min; SC: 10min. | **Direct cost savings for a cohort over 1 year:** -IV vs. SC administration: hospital saving of USD 650,710.94 in favor of SC = avoiding surgery to implant catheters (USD 451,799.69, 69%), preparation time (USD 180,161.56, 28%) and consumables (USD 18,749.69, 3%). | |
| Jackisch et al., 2015 (Jackisch et al., 2015) | Canada, Denmark, France, Russia, Spain, Switzerland | Prospective study [PrefHer (NCT01401166)] | HER2-overexpressing breast cancer | Trastuzumab | -Neoadjuvant chemotherapy followed by SC trastuzumab (600 mg) for 18 cycles followed by IV (standard dosing) compared with the reverse; -Cohort 1: SC by injection device; -Cohort 2: SC by handheld syringe. | -HCP time (preparation and administration); -Patient infusion chair time. | 415 | **Active HCP time - IV versus SC (preparation and administration):**  -Denmark: 7.2h versus 4.9h (-2.3h in favor of SC); France: 9h versus 5.7h (-3.3h in favor of SC); Canada: 11.8h versus 6.3h (-5.5h in favor of SC); Russia: 9.9h versus 5.2h (-4.7h in favor of SC); Spain: 8.2h versus 4.0h (-4.2h in favor of SC); Switzerland: 10.5h versus 7.2h (-3.3h in favor of SC).  **Chair time:**  Denmark: 78min versus 24 min (-69% in favor of SC); France: 85min versus 27min (-68% in favor of SC); Canada: 77min versus 24min (-68% in favor of SC); Russia: 47min versus 13min (-73% in favor of SC); Spain: 100min versus 20min (-80% in favor of SC); Switzerland: 133min versus 38min (-71% in favor of SC). | NR | |
| Rojas et al., 2020 (Rojas et al., 2020) | Chile | Model-based cost-minimization analysis study | HER2-positive early breast cancer | Trastuzumab | -SC: 600 mg fixed dose; -IV: loading dose of 8mg/kg and subsequent doses of 6 mg/kg 18 triweekly dosing cycles (1 year of treatment/a full treatment cycle) | -Preparation and administration costs; -Adverse drugs reactions treatment costs; -non-medical costs. | 100 | NR | **Preparation costs:** -Preparation costs/patient (single dose): IV: USD 3,481.75; SC: USD 4,069.08; cost-savings of USD 587.30/patient -Preparation costs for 1 year (18 doses): IV: USD 7,807,635.40; SC: USD 7,322,545,60; cost-savings of USD 485,089.87 -Main contributor: drug costs. **Administration costs (1 year = 18 doses):** -IV: USD 348,546.00; SC: USD 200,536.00; cost-savings of USD 148,010.00 in favor of SC; -Main contributors to the difference: costs of consumables, chair time, and the work time of the HCP). **Adverse drug reaction (ADR) treatment costs:** -IV: USD 157,425.00; SC: USD 171,537.50; cost-savings of USD 14,112.60 in favor of IV. -Serious ADRs associated with IV and SC are rare and have a low relative impact on overall costs. **Non-medical costs:**  IV: USD 8,330,911.10; SC: USD 7,706,775.80; cost-savings of USD 624,135.30 in favor of SC. **Total cost:** **IV: USD 8,330,911.10. SC: USD 7,706,775.80; cost-savings of USD 624,135.30 in favor of SC.** | |
| O'Shaughnessy et al., 2021 (O’Shaughnessy et al., 2021) | 16 countries | Randomized, open-label, international, multicenter, crossover, phase II [PHranceSCa (NCT03674112)] | HER2-positive early breast cancer | Pertuzumab and trastuzumab | Loading doses: -IV: P IV 840 mg; H IV 8 mg/kg; -SC: PH FDC SC 1200 mg P/600 mg H in 15 mL. Maintenance doses: -IV: P IV 420 mg; H IV 6 mg/kg;  -SC: PH FDC SC 600 mg P/600 mg H in 10 mL. | -HCP time (preparation and administration); -Patient infusion chair time. | 80 (in each group) | **Median patient time:** -SC: 33.0 to 50.0 min; IV: 130.0 - 300.0 min. **Median administration:** -SC: 7.0 - 8.0 min; IV: 60.0 - 150.0 min. | NR | |
| HER2: human epidermal growth factor receptor 2; SC: subcutaneous; IV: intravenous; T&M: time and motion; SID: single-use injection device; HHS: handheld syringe; HCP: healthcare professionals; NR: not reported; P: Pertuzumab (Perjeta); H: trastuzumab (Herceptin); P+H IV: intravenous pertuzumab plus trastuzumab; PH FDC SC: fixed-dose combination of Perjeta and Herceptin for subcutaneous injection. | | | | | | | | | | |
| *PrefHer trial: switch from SC to IV or vice versa | | | | | | | | | |  |
| **all values are expressed as mean. When the study provides data in another unit, the unit was mentioned, i.e., median. | | | | | | | | | | |
| ***currency was standardized in United States Dollars (USD) on March 27, 2023. | | | | | | | | | | |
